# What Constitutes a Phrase in Sound-Based Music? A Mixed-Methods Investigation of Perception and Acoustics

**DOI:** 10.1371/journal.pone.0167643

**Published:** 2016-12-20

**Authors:** Kirk N. Olsen, Roger T. Dean, Yvonne Leung

**Affiliations:** 1 The MARCS Institute for Brain, Behaviour and Development, Western Sydney University, Sydney, New South Wales, Australia; 2 Department of Psychology, Macquarie University, Sydney, New South Wales, Australia; University of Zurich, SWITZERLAND

## Abstract

Phrasing facilitates the organization of auditory information and is central to speech and music. Not surprisingly, aspects of changing intensity, rhythm, and pitch are key determinants of musical phrases and their boundaries in instrumental note-based music. Different kinds of speech (such as tone- vs. stress-languages) share these features in different proportions and form an instructive comparison. However, little is known about whether or how musical phrasing is perceived in sound-based music, where the basic musical unit from which a piece is created is commonly non-instrumental continuous *sounds*, rather than instrumental discontinuous *notes*. This issue forms the target of the present paper. Twenty participants (17 untrained in music) were presented with six stimuli derived from sound-based music, note-based music, and environmental sound. Their task was to indicate each occurrence of a perceived phrase and qualitatively describe key characteristics of the stimulus associated with each phrase response. It was hypothesized that sound-based music does elicit phrase perception, and that this is primarily associated with temporal changes in intensity and timbre, rather than rhythm and pitch. Results supported this hypothesis. Qualitative analysis of participant descriptions showed that for sound-based music, the majority of perceived phrases were associated with intensity or timbral change. For the note-based piano piece, rhythm was the main theme associated with perceived musical phrasing. We modeled the occurrence in time of perceived musical phrases with recurrent event ‘hazard’ analyses using time-series data representing acoustic predictors associated with intensity, spectral flatness, and rhythmic density. Acoustic intensity and timbre (represented here by spectral flatness) were strong predictors of perceived musical phrasing in sound-based music, and rhythm was only predictive for the piano piece. A further analysis including five additional spectral measures linked to timbre strengthened the models. Overall, results show that even when little of the pitch and rhythm information important for phrasing in note-based music is available, phrasing is still perceived, primarily in response to changes of intensity and timbre. Implications for electroacoustic music composition and music recommender systems are discussed.

## 1. Introduction

Phrasing is important for structuring auditory streams and facilitates the organization of auditory information [1, 2]. Speech and music are two domains where phrasing is commonplace, though the descriptors used are very different. In speech, a phrase constitutes a low level of a complex hierarchy. A phrase usually comprises a few words that taken together in larger clauses can constitute meaning, most commonly in the form of a sentence. To determine boundaries between segments of any kind in speech (notably between words, clauses, and sentences), infant and adult listeners use acoustic cues such as changes in intensity/amplitude, rhythmic (durational) pattern, and pitch contour [3–5]. In note-based instrumental or vocal music, pitch-related aspects of the music are primary determinants of how listeners perceive and segment a musical phrase; for example, contrasts of pitch range and changes in melodic contour and tonal stress [1, 6–9]. As in speech, the importance of pitch-related aspects of music for segmentation is observed at very early stages of development: infants six-months of age prefer to listen to segments of music delineated by a drop in pitch height, a segment-final increase in pitch duration in a melody, and a predominance of octave simultaneities [10]. The binding of instrumental pitched (and unpitched percussive) notes to rhythmic structure is also important in note-based music. That is, given a clear note energy envelope of attack, sustain, and decay, individual notes are separable and delineate rhythms associated with musical phrases.

However, contrast the case of 'sound-based' music, a category description developed by professional music analysts, composers, and improvisers. Sound-based music has been important for at least the last 60 years [11]. In characterizing sound-based music, Landy [12] recognizes a continuum between it and note-based music. At one extreme, for example, a rhythmic instrumental pop-song or Beethoven's Waldstein Sonata for piano constitutes note-based music. These pieces are characterized by discrete events with onset attacks, decays, offsets, clear rhythmic patterns, and pitch-based melodies and harmonies that are generally realized by human performers. At or near the other extreme fall noise music, much electroacoustic music, and sound art (all illustrated in the works included in the present study). There may be few if any discrete events in sound-based music; that is, events separated by clear acoustic offsets and onsets with nothing intervening. Instead, sound continua are commonplace and often intended as acousmatic music (i.e., music for realization by loudspeakers rather than performers) and sometimes largely or entirely computer generated [13]. Consequently, there may be few transparent rhythmic repetitions and pitch may be essentially absent, though both can be readily asserted (as commonly exploited in electronic dance music). From a musician's compositional and analytical point of view (but not necessarily the point of view of untrained listeners), the focus of such music moves from controlling changes in pitch, harmony, rhythm, and intensity, to changes in timbre and intensity. There are numerous intermediate forms. For example, some glitch music creates obvious recurrent rhythms from digital artefacts superimposed on sound continua. Similarly, the genre of ‘drum and bass’ often comprises a sound continuum in conjunction with rhythmic percussive and bass frequency patterns, the latter sometimes not articulated with the sharp attacks and decays that occur in most instrumental music. Speech (and less so singing) is also intermediate form, and its digital transforms are commonly used in electroacoustic sound-based composition [14]. The sound sources most fundamental to biology, those of the environment, also normally fall into this intermediate sound-based continuum, and we include an example of such sound in our study.

Given this theoretical contrast between note- and sound-based music, we investigate here whether phrasing in sound-based music can be perceived by untrained listeners, and if so, what might be the structural elements important for such perception of musical phrasing when common pitch and rhythmic aspects of note-based music are removed or reduced, at least from the perspective of the music creators. What we know about note-based music’s formal description and the perception of its musical phrasing may not translate to sound-based music. In the presence of limited pitch and rhythmic information (unlike note-based music), we propose that additional acoustic attributes such as changes in timbre may join with changes of intensity to explain listeners’ perception of phrases in sound-based music. We do not anticipate that timbre and intensity are uninvolved in phrasing in note-based music; they do impact note-based phrasing in both tonal and atonal music [6, 7, 15]. Rather, we hypothesize that timbre and intensity dominate over pitch- and rhythm-related attributes in sound-based music in particular.

Although most listeners are unfamiliar with sound-based music [16], there is strong evidence that listeners unfamiliar with a particular musical style are still able to understand and extract important structural aspects [17]. This is especially evident in the context of cross-cultural expression of emotion in music. Listeners unfamiliar with certain culture-specific styles of music are able to successfully extract emotional meaning through acoustic cues common across cultures, even though specific culturally determined cues and conventions are not initially available to them [17, 18]. We suggest that a similar mechanism applies here: although listeners unfamiliar with sound-based music will not necessarily immediately understand or extract the genre-specific conventions of such music, they will nevertheless use available acoustic cues common to most genres (and to environmental or speech sounds) to perceive and segment the unfamiliar music; specifically, timbre and intensity. A now extensive body of work investigating the acoustic factors that predict affective responses to substantial extracts (2–3 minutes in duration) of several pieces of sound-based music supports this view (e.g., [16, 19–22]). In this context, therefore, we will now address what little is already known about perceptual segmentation of sound-based music. We will then review relevant research regarding perceptual segmentation of speech. As mentioned, speech segmentation is considered here in the context of sound-based music because speech is a congener of sound-based music, is widely experienced as environmental sound, and directly informs the design of our study.

### 1.1. Perceptual segmentation of sound-based music

The perceptual formation of multiple streams within an ongoing sound structure is often termed auditory scene analysis [2]. Specifically, it is known that the formation of continua between tones (such as tones in two different pitch registers) makes it more difficult for listeners to segregate streams of auditory information [23]. Segregation is commonly influenced by both static and dynamic aspects of acoustic spectra (e.g., with instrumental timbres, native and modified) [24, 25]. Such segregation data suggest that our sound-based continua may be segmented less frequently than note-based music, and that both static and dynamic spectral aspects may again contribute. Therefore, we aim to detect segmentation amongst successive components of a sound-based piece of music, whether there are multiple sonic streams or not.

There have been only a few experiments investigating the dynamic elements important for event segmentation of sound-based music. For example, Bailes and Dean [26–28] used directly apposed and briefly cross-faded pairs of short segments of sound (5 or 11 seconds in duration). They found that listeners were able to perceive segments comprising noise, sine-waves, ‘busy’ sounding electronic segments, chimes, water, and drum loops when the boundaries of perceived segments were characterized by abrupt changes in acoustic intensity (experienced perceptually as loudness; for a review, see [29]) and spectral flatness (a measure closely related to perceived timbre [28]). There was an asymmetry in boundary perception, in that a pair of sounds were well segmented when the intensity increased or when a frequency band was added, but not vice versa; a result consistent with the effects of auditory masking [30, 31].

In [14], a range of excerpts including some sound-based pieces, each of the order of two minutes, was studied (with mostly untrained listeners) for implicit perceptual segmentation by taking a continuous measure of perceived change and using statistical 'change-point' analysis to determine segments. This analysis primarily delineates when a segment (in this case of a time-series of perceived change) differs from another, within defined statistical limits, and not simply a point of change as its name somewhat misleadingly implies. The results showed that segments defined from a musicological point of view, and in some cases on the basis of changing human agency, coincided quite closely with those detected in the continuous perceptions for sound-based, note-based, and poetic artificial-language speech stimuli [14]. Acoustic features that might predict this segmentation were not investigated.

In another interesting approach to the question of perceptual segmentation of sound-based music, a 38-second section of an electroacoustic piece, *Ciguri* by Chilean composer Felipe Otondo, was presented to 22 participants, 81% of whom were music students or lecturers (i.e., trained musicians) [32]. This piece falls into an intermediate grouping between the sound- and note-based music definitions above, mainly because it has an insistent and virtually isochronic rapid percussion attack, together with one or more streams of sustained electroacoustic sound with somewhat clear pitch structure. Participants pressed a button when they perceived a change in the music. Listeners varied from perceiving 0 to 16 segments, with a mean of 3.9 (i.e., a mean duration of about 10 seconds). Kernel density estimation was used to produce a single sequence of data representing the frequency with which the whole group of participants detected a change. This sequence was interpreted as representing a consensus of six segments. The study segmented the piece computationally for comparison with this. Fast Fourier transforms (FFT) of the audio signal (sampled at 20Hz) were used to compute a self-similarity matrix from which a 'novelty' measure across the series was obtained. The resulting sequential distance measures were used to detect peaks that were subsequently taken to be points of segmentation. These methods have been discussed in detail in relation to annotation of acousmatic music [33]. Garay [32] found considerable similarity between the perceived and computed measures, and semantic analysis suggested important roles of perceived loudness, pitch, and timbre for perceptions.

In a follow up experiment using a stimulus comprising a single-stream sequence of concatenated everyday sounds, but with only 33% of participants being music students or lecturers, participants' perceptions were interestingly closer to the computational segmentation in [14] than to the concatenation points, even though little attempt was made to smooth the concatenation of the sounds. The computational measure, while using the whole spectrum of the sound by FFT, was not designed in terms of specific spectral components nor to identify acoustic predictors of perceptions.

Although computational annotation of acousmatic music has been successful in defining segments detected by analysts, or sometimes prefigured by composers [33], it has to be noted that there is a substantial lack of data on such music (i.e., perceptual responses by a significant number of individuals, whether expert or not). Consistent with this, a recent paper reviews the disparities in outlook and purpose (even towards timbre) between music psychology and other fields, and suggests approaches to bring them together productively [34]. The present study begins to address such disparities by investigating predictors amongst acoustic factors, particularly spectral features, which might model and potentially explain listeners' perceptions. We also focus on the majority of musical audiences: people without specific musical training.

### 1.2. Perceptual segmentation of speech and its relevance to sound-based music

Knowledge of language learning in the speech domain can inform our investigation of perceptual segmentation of sound-based music and musical phrasing. A musical phrase, as used in musicology and delineated in our instructions to participants (see below), is a grouping of multiple distinguishable events, be they notes or sounds, discrete or continuous. The closest analogous units of speech, in which we are almost all expert, are clauses and sentences rather than the usually much shorter linguistic phrases. The vast literature on perception of segments in speech across languages largely focuses on learning and recognition of words, rather than larger units, and on cross-linguistic aspects of language learning across the lifespan (for an in depth review, see [35]). Languages are commonly classified into stress- and tone-languages [35, 36]. In stress languages, units such as words are distinguished from each other to a considerable extent by the patterns of acoustic intensity they contain. In tone languages, a much greater role is played by changes in fundamental frequency (the lowest frequency energy peak in the speech entity spectrum, commonly described as 'pitch') and sometimes consistent relative changes in the first formant and higher partials.

A listener confronted with unfamiliar music from, for example, a culture other than their own or from sound-based music (assuming as normal that their prior exposure was primarily to note-based music), is in a position of inexperience somewhat related to a baby learning its native language, or more closely, an adult learning a second language; especially those learning a tone-based language whose primary language is stress-based, or vice versa. Such an unfamiliar musical listener may closer represent an adult learning a second language because they may have gained fluency in cognizing key features, but at the expense of cognizing contrasting features that are more important to the new kind of music. This process in speech learning is sometimes termed perceptual 'desensitization' [37, 38].

So what does our knowledge of the learning of language and speech suggest about the learning and segmentation of sound-based music and musical phrasing? First, studies of artificial languages (constructed from phonemic units similar to those of a genuine language, but concatenated without forming genuine words) or artificial phoneme contrasts reveal that learning aspects of the segmentation of languages can be very fast, sometimes occurring even within two minutes [39]. Second, such studies suggest that a few features are very important for the learning of word-boundaries. A detailed review of infant language development [40] points out that spoken words run into each other, blurring word boundaries; a blurring, we argue, that may be similar to the continuum between note- and sound-based music. Once our native language is learnt, we begin to sustain the illusion that boundaries are clear [35]. Learning the segmentation in the first place (often described as the 'bootstrapping problem') is largely dependent on developing relative weightings for the relevance of pause duration, pitch, and pre-boundary lengthening [40, 41]. Pause does not refer to silence, but rather the relative length of time between increments of acoustic intensity. Pre-boundary lengthening refers to the fact that the length of the smallest phonemic units comprising a word is often greatest for the last. Note that in acoustic terms applicable to music, these factors could be considered, respectively, primarily as a pause in event activity, a timbral flux (the 'pitch' component), and a temporal elongation of a particular timbral element. In each case, these are delineated in conjunction with changes of acoustic intensity.

Successful computational speech segmentation based on intensity temporal profiles within narrow frequency bands across the spectrum [42] supports the suggestion that it is multiple frequency components of speech that are important (i.e., timbre) and not just 'pitch' per se, because pitch is a single dimensional representation of a sound, even when determined by (multi-dimensional) spectral patterns [43]. Statistical learning of adjacent and non-adjacent feature-dependency is important [44, 45], but infants probably cannot solely rely on this to ‘bootstrap’ [40, 46, 47].

How do these aspects of learning word boundaries contribute to learning linguistic phrases, clauses, and sentence segmentation and their hierarchies? Johnson [40] emphasizes that an infant learns segmentation with respect to all linguistic units in parallel, with progressive acquisition of each ability. However, at the level of larger syntactic units such as clauses, the three features just emphasized (pause duration, pitch, and pre-boundary lengthening) now contribute to speech 'prosody' together with more substantial variations in acoustic intensity. Such prosody often systematically delineates the larger syntactic units and their hierarchies [48]. Again, prosodic cues are weighted [41] and this can vary between individuals and languages [49]. The 'Edge Hypothesis' has been developed to explain universal cues to word boundaries delivered by these features, but with reference not only to words, but also speech phrases, clauses, and sentences [50].

Even this brief summary of the speech literature indicates that besides pitch, timbre, and intensity, an investigation of phrase structure in music needs to emphasize the consideration of silences, or at least 'pauses' in activity characterized by decreased acoustic intensity and less frequent small events. In the case of sound-based music, we cannot readily estimate the frequency of small events (given they are presently undefined), and so pauses are represented in the intensity flow and particularly in the extent of variation relative to its mean value. We include this parameter in our analyses. For a more in depth review of speech-music parallels, see [36].

### 1.3. Acoustic features of timbre in sound-based music

To investigate the role of intensity and timbre in listeners’ perception of musical phrases in sound-based music, we first measured acoustic intensity as a global proxy for perceived loudness, and spectral flatness as a global proxy for perceived timbre. However, numerous additional spectral parameters have previously been linked to the elusive notion of ‘timbre’ [51, 52]. Therefore, a more detailed timbral analysis was also conducted here using a range of additional parameters based on the recommendations in [53], where development and application of a Matlab Timbre Toolbox for investigating perception of dissimilarity between pairs of short instrumental sounds is described. The stimuli studied in [53] were thus in dramatic contrast to those of the present sound-based music, so the recommendations of the paper are treated cautiously here. Nevertheless, we followed the general suggestion to use a measure of both central tendency and temporal dispersion of each descriptor, all of which are here time varying. As a result, the parameters related to timbre that were used in our additional analysis of perceived musical phrasing in sound-based music were spectral centroid, spectral flux, spectral spread, inharmonicity and roughness. These were the features which showed inter-correlations of < .5 in previous analyses of instrumental sounds [53].

### 1.4. Aim, design, and hypotheses

The aim of the present study was to investigate whether musically untrained listeners could perceive phrases in the varied music presented, and if so, to determine the structural elements important for perception of musical phrasing in sound-based music in particular; music where pitch and rhythmic aspects as enunciated in instrumental note-based music are removed or reduced and transformed. The work in [26–28] showed that specific acoustic parameters related to changes in loudness and timbre can elicit perceived segmentation, even when no familiar note-based cues were present. However, the segments in their stimuli were temporally predefined on each occasion to either 5 s or 11 s. In the present study, we do not predetermine phrase durations, but rather, allow the listener to choose when a phrase has ended. It is nevertheless desirable to define the minimum temporal window for phrase perception in our study. To this end, recent work on the acoustic influences of continuously perceived affect has shown that such influences operate over periods of approximately five seconds and longer [16, 19–22, 54]. Taken together with the work of Bailes and Dean [26–28], this suggests a temporal window of five seconds as an appropriate minimum duration to address perceived musical phrasing in sound-based music. Furthermore, for perceived phrases longer than five seconds, acoustic attributes associated with the final five seconds of a perceived musical phrase may be disproportionally influential; a kind of ‘recency’ effect that commonly explains a memory recall advantage for the most recent item in a series of items [55]. Therefore, we investigate the importance of acoustic intensity and spectral parameters for listeners’ perception of musical phrases by analyzing: (1) the acoustic content across each entire perceived phrase (a whole-phrase ‘global’ approach); and (2) the acoustic content comprising the final five seconds of a perceived phrase (a ‘terminal portion’ approach).

We used a mixed methods approach with a range of auditory stimuli comprising sound-based music, note-based instrumental music, and environmental sound (see Methods section for more detail). Participants made qualitative descriptions of each perceived musical phrase throughout each stimulus, and timings reflecting the onset and offset boundaries of each perceived musical phrase were measured. Thematic analysis of qualitative data led to three categories describing the most salient aspect of all perceived phrases: ‘Intensity’, ‘Timbre’, and particularly in the case of the instrumental note-based music we studied, ‘Rhythm’. These qualitative descriptions for each perceived phrase are presented in the tables in the Supporting Information file (S1 Appendix). Analysis of acoustic features associated with these three categories was carried out to extract time-series data for intensity, spectral flatness (timbre), and rhythmic density (for the instrumental note-based stimulus). These acoustic time-series data were then used to model listeners’ perception of musical phrases both globally and also on a phrase category-by-category basis. In a subsequent analysis, several other spectral parameters (spectral centroid, spectral flatness, spectral spread, inharmonicity, and roughness) were assessed as possible additional predictors of phrase perception. Specifically, it was hypothesized that:

1. Sound-based music and environmental sound does elicit phrase perceptions, and these are primarily associated with temporal changes in acoustic parameters of intensity and timbre.
2. Temporal change in rhythmic pattern and rhythmic density is a major factor in modeling listeners’ perception of phrases in instrumental note-based music.
3. Pauses in the continuity of music are important in segmentation of sound-based and note-based music, as they are in speech.

## 2. Method

### 2.1. Participants

Twenty adult psychology students were recruited from the University of Western Sydney (17 females and 3 males; *M* = 21.84 years, *SD* = 5.42, range = 18–41 years). Three participants had received individual musical training (*M* = 5.67 years, *SD* = 5.69, Range = 1–14 years). All reported normal hearing. This research was conducted according to the principles expressed in the Declaration of Helsinki and approved by Western Sydney University’s Human Research Ethics Committee (approval #H9869). All participants provided written informed consent.

### 2.2. Stimuli and equipment

Stimuli comprised six pre-recorded excerpts that ranged amongst sound-based music, instrumental note-based music, and environmental sound. The rationale for selection was to contrast sonic material that comprised no obvious instrumentation, a hybrid combination of sound sources, and note-based instrumental music. A brief analytical/compositional description of each stimulus is provided below:

1. BBC SoundFX CD20 Weather(1) (1989) *‘Tree Creaking in Strong Wind’* (2’20”): This excerpt is from a field recording of wind moving through trees (it is not a human 'composition'). The overall characteristics of the excerpt resemble noise, but in a naturalistic context.
2. Martin Ng and Roger Dean (2000) *‘LowHz’* (2’13”): A noise-based piece excerpted from an immersive real-time computer improvisation using audio software MAX/MSP (Cycling 74, San Francisco). A complete version is on compact disc in Dean [56].
3. Brian Eno (1992) *‘Francisco’* (2’20”). This piece is primarily ambient in its composition with little obvious human agency or identifiable sound source.
4. Trevor Wishart (1977) *‘Red Bird*, *a political prisoner’s dream’* (2’17”): This was excerpted from a recording on UbuWeb of this 45 min piece for tape. It has a strong narrative of hybrid combinations of human and animate sounds.
5. Ludwig van Beethoven (1804) ‘*Sonata No*. *21 in C major*, *Op*. *53 Waldstein’* (the opening 2’26”). This involves a single note-based musical instrument (piano) with obvious human agency and urgent repetitive rhythmic drive.
6. Iannis Xenakis (1955) *‘Metastaseis’* (1’59”). An orchestral piece with multiple instruments and apparent human agency. Although this excerpt involves instruments, it is characterized by multi-instrument sound clusters and large glissandi that make it sound closer to noise-based music than prototypical orchestral note-based music. The excerpt mostly lacks clear repetitive rhythms.

Practice trials used two additional stimuli: excerpts of the first movement from Mozart’s *‘Symphony No*. *40’* (1’18”) and of Xenakis’s electro-acoustic piece *‘Orient-Occident’* (1’29”). Each stimulus excerpt in the experiment was presented as an.aiff stereo 16 bit audio file with a 44.1kHz sampling rate. The pieces 1–6 were always presented in the sequence listed above to achieve a gradation from sound based items to instrumental music and then from piano to orchestra, as well as to establish a positive gradient of the extent of human agency likely perceived in the performances. This was designed to minimize a continuing focus on roles of obvious human agency or physical source origins in the sound-based test stimuli. Given the relatively long duration of the stimuli and the considerable familiarization that would likely occur across the time-course of the experiment, a conventional counterbalanced order would not have homogenized the responses. The experiment was conducted in a sound attenuated booth and stimuli were presented binaurally through Sennheiser HD25 headphones. An Apple MacBook Pro laptop computer (System 10.6.2) using a custom written program in MAX/MSP (Cycling 74, San Francisco) software displayed on-screen instructions and response buttons, presented stimuli, and continuously recorded data.

### 2.3. Procedure

Participants first read an experiment information sheet, gave written informed consent, completed a brief demographic questionnaire, and then received standardised instructions regarding the experiment. Participation was divided into two tasks per stimulus: (1) a continuous phrase-task response; and (2) an intermittent phrase-detection task including qualitative feedback. The continuous phrase-detection task required participants to press the ‘space-bar’ on the computer keyboard each time they perceived an end of a phrase. As the sample mainly comprised non-musicians, we did not wish to assume that participants would understand the concept of a musical 'phrase'. Therefore, we instructed participants to respond to ‘events’ rather than ‘musical phrases’, but used examples from speech to define what was meant by an ‘event’ in the experiment. Specifically, the instruction was: “…when listening to each excerpt of music, indicate the times throughout the music where you perceive the end of an event”. We elaborated further on how to conceptualize an event: “You can think of an event to be a little bit like a sentence or clause in day-to-day speech, but in this experiment it is in the context of non-speech auditory material. For example, events have a beginning, they occur over different durations of around five seconds or even longer, will comprise pieces of auditory information that may vary in different ways throughout the event, and of course, will have an ending.” These examples were intended to clarify that potentially perceived events were not to be considered in extremely short time-frames, such as individually sounded notes in note-based music, but rather, relatively longer time frames that in musicological terms coincide more closely with musical ‘phrases’ than musical notes.

When each response was made in the continuous phrase-detection task, the stimulus continued to play until completion, hence the use of the label ‘continuous’ phrase-detection. Each response was time-stamped and constituted the end of one perceived phrase. Thus, the beginning of the next phrase was time-marked as 1 ms after each button-press response. A 1kHz pure tone was presented within each stimulus at a set time between 10–20 s after stimulus onset (randomly determined for each stimulus for all participants before commencing the experiment). Participants were required to make their first button-press response when they heard the pure tone. For analytical purposes, this first response marked the beginning of the first phrase, the end of which was determined by the participant’s next response. The duration between stimulus onset and the pure tone was designed to allow enough time for participants to become aware of the style and content of each stimulus before making their responses. This ‘orientation’ period was important because the stimulus set comprised varied unfamiliar musical genres and complexities.

Once participants completed the continuous phrase-detection task for one stimulus, the same stimulus was then repeated in the following trial, but with an intermittent phrase-detection task. In this second task, the participant made a button-press response indicating they had perceived the end of a phrase, upon which the music stopped, a text-box appeared on the computer monitor, and the participant was instructed to: “please use the keyboard to describe in a sentence or two: what was happening in the music that made you perceive the end of an event? In other words, try your best to write out the reasons why you made your response at that particular point in the music.” Once the participant had made their qualitative response, they clicked a ‘continue’ button and the music continued playing from the point at which it had stopped. The resulting data thus included a series of time-stamped responses for each stimulus combined with qualitative data describing the characteristics of each response.

We expected there to be familiarisation during the first task (which might enhance sensitivity to phrase detection), but we also expected the more extensive demands of the second task to restrict the number of phrases a participant would register, whether because of a wish to make the task as easy as possible or because of the need for self-justification of each decision. Thus we had no predictions as to the relative number of responses in first versus second tasks, but we did expect responses in the second task to mostly reflect those that occurred during the first. Because ‘task’ was not intended as an independent variable in our experiments, we merely present simple descriptive statistics relating performance between both tasks (see Results below).

Once the continuous and intermittent event-detection tasks were completed for one stimulus, the next stimulus was presented and the two tasks were completed again, and so on until all six stimuli had been presented. Therefore, each stimulus was presented twice in the experiment for a total of 12 trials (not including practice), with two tasks completed for each stimulus. For statistical modeling presented below, only responses from the second task are presented because they contain the onset/offset time-stamps for each perceived phrase in addition to qualitative data describing each response. Overall, the experiment took approximately one hour to complete.

### 2.4. Acoustic analyses and statistical approach

Three acoustic measures relevant for the three main features of intensity, timbre, and rhythm (in the case of the Beethoven *Waldstein Piano Sonata*) were obtained by means of acoustic analysis. First, the intensity (dB SPL; sound pressure level measured in decibels) and spectral flatness (Wiener entropy) profile of each stimulus was obtained using Praat (version 5.3.23) at a 2Hz sampling rate. These measures have been detailed previously [16, 19]. For rhythm in the *Waldstein Piano Sonata*, the number of acoustic note onsets was calculated and summed within 500 ms windows to give an indication of the change in rhythmic density (or sparsity) that occurs (this was achieved with the aid of Sonic Visualiser, but careful adjustments and additions to the events identified by the several note onset detection algorithms were required). Five additional spectral parameters related to timbre, as explained above, were also measured for use in more elaborate statistical models of perceived phrasing. These were calculated using MAX/MSP (Cycling 74, San Francicso) with the zsa package (spectral centroid, flux, spread), the CNMAT (University of California, Berkeley) patch for roughness, and Alex Harker's package for inharmonicity.

From each such continuous parameter, three unitary predictors were derived for each phrase under analysis, consistent with the recommendations in [53]. Note that phrases and corresponding predictors remained defined on an individual participant basis; in other words, phrase start and end times were not averaged across participants. The three derived predictors were: the mean value, the mean of its differenced values across a phrase (a representation of the overall magnitude and direction of rate of change) and the mean of the absolute values of that differenced series (a representation of the variability of the rate of change). For the single case in which rhythm was strong (the Beethoven *Waldstein Piano Sonata*), we treated the rhythmic density series in the same way as the other predictors. Thus, there were three potential predictors derived for each of the features of intensity, timbre, and rhythm. For Beethoven’s *Waldstein Piano Sonata*, we also considered 'gaps' (or extreme sparsity) in the note stream as predictors of perceived phrases. For convenience, we refer to these factors generically below as 'acoustic' features, even though the rhythmic feature is arguably musical.

For each stimulus, the overall perceived phrase timing series represent a 'clustered recurrent event process' [57]. In essence, this means that across participants the series of times characterising phrases are not simply randomly dispersed in time (as in a Poisson process), but clustered around particular successive times (as can be seen in the results). Therefore, the influence of the three main acoustic features on participants’ perception of phrasing can be modelled using variants of ‘survival analysis’ based on Cox hazard modeling [58]. Survival analysis is typical in modeling the expected amount of time until one (e.g., death of an organism) or a series of events occur, most commonly in biological or mechanical systems. Recurrent events may be considered as either failures as a result of 'frailty' (such as succumbing to an infection) or successes as a result of 'achievement' (such as here identifying that a phrase has ended). The fact that each individual participant has repeated ‘successes’ through successive phrase identifications means that each response cannot be assumed to be independent. We allow for this by using a 'cluster' procedure that provides robust standard errors for overall models.

The original Cox procedure [58] utilised 'proportional hazards': the hazard change (i.e., the change in the 'risk' of the event occurring) was assumed to bear a constant linear relation to the ordinal and continuous predictors: an *n-*percent change in a predictor caused a fixed-percent change in the value by which the hazard (risk) differed from its baseline value. We use developments of the model that permit time-dependent coefficients upon these predictors by means of the 'Survival' package in *R* (version 3.2.2) with algorithms coded in the *RStudio* development environment (version 0.99.491), mainly involving the core ‘cox.ph’ function.

Modeling was undertaken in a defined procedure. For the initial models of all perceived phrases, allowed predictors were the three forms (defined above) of averaging intensity and spectral flatness time series, and the rhythm density series for the Beethoven’s *Waldstein Piano Sonata* stimulus. Up to two time transformed predictors (abbreviated ‘tt’ in the Tables) were allowed, either a linear x*t or a logarithmic x*log(t) transform: no attempt was made to optimise these further. The transform simply multiplies the predictor variable ‘x’ by the time function, and the model optimises the coefficient of the resulting derived predictor. The predictors to be tested as time transforms were chosen as those with the largest rho parameter in the Survival package zcox.ph function, which are indicative of the largest variations with time. Models were selected for parsimony by removing all variables that were not individually significant, unless the removal caused a total failure of the model (two cases only, displayed with an asterisk in Table 4 of the Results). With the exception of these two cases, this also resulted in optimising the models’ Bayesian Information Criterion (BIC), a stringent assessment of parsimony that strongly penalises for the addition of predictors.

For separate models of perceived phrases categorised by timbre, intensity, or rhythm, exactly the same procedure was used. Note there is a limitation that the resultant time series do not contain all the data, and hence may not be fully representative of the original series, some of whose autocorrelations may be disrupted. Without time-dependent transforms, the models described so far commonly showed modest *R*^2^ values with fits of between .2 and .3, but these were often improved with time-dependence (*R*^2^ values between .35 and .5).

In the final analyses, we assessed the possible roles of five additional spectral predictors in the whole-phrase perception models. These predictors were also used in the three forms just described (mean, mean change, and mean absolute change on phrase by phrase and individual by individual basis). This resulted in models with up to 23 predictors. We limited the search path required to select amongst the possible models for parsimony by means of additional constraints: the starting model was always taken as the simpler model defined earlier (with only spectral flatness amongst the considered timbral predictors), plus the 15 new spectral predictors. This was refined by removal of individually non-significant predictors, after which the alternative time transforms were assessed. Finally, alternative interaction parameters were also considered on the basis of the most substantial predictors. Note that the possible value ranges of the spectral predictors varied and hence, their impact in the model depended not only on their coefficients.

## 3. Results

### 3.1. Descriptions of perceived musical phrases

**Fig 1:**
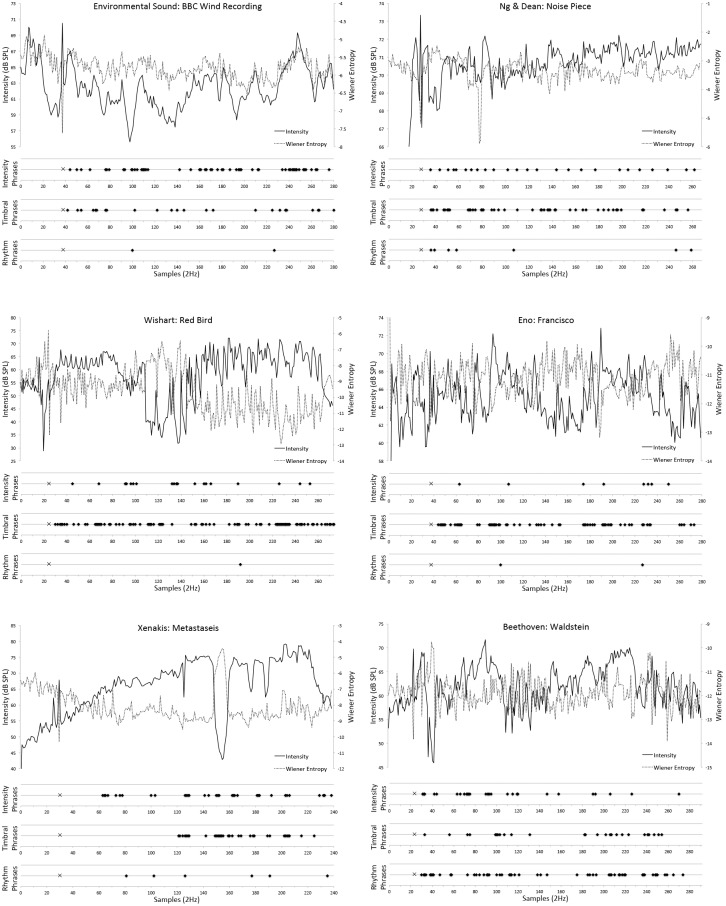
Time-stamped phrase responses and acoustic time-series data. Displays all time-stamped responses assigned to timbre, intensity, or rhythm categories across the entire time-course of all six stimuli. Acoustic categories were based on thematic analyses of participants’ qualitative descriptions of each perceived phrase. The diamond-shaped markers on the three horizontal scales in each panel signify these data and specifically, the point at which each phrase was perceived to have ended. The ‘×’ symbol signifies the moment in each stimulus where a 1kHz pure tone was presented. The pure-tone indicated to participants that they were to begin responding and was used to mark the beginning of the first perceived phrase. Time-series data of acoustic intensity (solid line; dB SPL on left y-axis) and spectral flatness (dashed line; Wiener entropy on right y-axis) are also plotted for each stimulus. All perceptual and acoustic data are presented at a sampling rate of 2Hz.

It was clear that every participant could readily detect phrases in all stimulus items, though varying in mean duration as expected. Furthermore, these perceptions were coherent across participants (as revealed by the clustering in Fig 1). The mean duration of perceived phrases across all pieces and participants was 15.05 seconds (as judged at the sampling rate of Fig 1, 2Hz). There were fewest phrases perceived in the BBC *‘Wind’* environmental sound stimulus (appropriately, given its lack of compositional design) and most in the Wishart *‘Red Bird’* and Beethoven *‘Waldstein’* stimuli, which can readily be viewed as the most variegated of the pieces. The experiment was not designed to generate consistency between frequency of responses in the first ‘continuous phrase-detection’ task and the second more demanding and informative ‘intermittent phrase-detection’ task, but we conducted a descriptive analysis of the similarity of phrase identification responses between both tasks (based on frequency of occurrence). Due to the different requirements of the two tasks and an increase in familiarity (by design) from prior exposure in Task 1, we did not expect or require a high level of agreement in the frequency of phrase detection between responses in Task 1 and Task 2 for each stimulus. However, the frequency of responses in Task 1 and Task 2 differed by less than 7% when considered relative to the total number of responses for each of the Ng and Dean *‘LowHz’*, Wishart *‘Red Bird’*, Eno *‘Francisco’*, and Xenakis *‘Metastaseis’* stimuli. The frequency of phrase detection responses in Task 2 for the Beethoven *‘Waldstein’* stimulus was 12.10% lower than the frequency in Task 1, and 19.34% lower in Task 2 relative to Task 1 for the BBC *‘Wind’* environmental sound stimulus. For the remaining analyses, we consider only the phrase detection data from the second ‘intermittent phrase-detection’ and qualitative description task.

Fig 1 displays six panels of time-series acoustic data in the form of intensity profiles (solid line; dB SPL on left y-axes) and spectral flatness (dashed line; Wiener entropy on right y-axes) for each stimulus. Furthermore, diamond-shaped markers on the three horizontal scales in each panel indicate all participants’ perceived phrase responses (quantized to 2Hz) across the time-course of each stimulus. From the process of thematic qualitative analysis, these responses were placed by the authors into categories of intensity, timbre, and rhythm, labeled as such in each of the three y-axis titles per panel. All qualitative descriptions of perceived phrases are presented in S1 Appendix. The number of responses in each category for each stimulus is presented in Table 1.

**Table 1:** Number of perceived phrases assigned to intensity, timbre, or rhythm categories.

Stimulus	Stimulus Description	Assigned Acoustic Category			
		Timbre	Intensity	Rhythm	Total
BBC SoundFX: *Wind*	Environmental sound	24	68	2	94
Ng & Dean: *LowHz*	Noise sound-based piece	68	25	7	100
Eno: *Francisco*	Ambient sound-based piece	95	8	2	105
Wishart: *Red Bird*	Hybrid sound-based piece	123	22	1	146
Beethoven: *Waldstein*	Instrumental note-based piece	32	31	64	127
Xenakis: *Metastaseis*	Instrumental sound-based piece	45	49	6	100

**Note.** Categories assigned to each perceived phrase across participants were based on thematic analyses conducted by the authors of participants’ qualitative descriptions of each perceived phrase (see S1 Appendix).

As can be seen in Fig 1 and Table 1, the majority of perceived phrases for the BBC *‘Wind’* environmental sound stimulus (comprising primarily of wind rushing through tress) were associated with intensity. For the Ng and Dean *‘LowHz’*, Wishart *‘Red Bird’*, and Eno *‘Francisco’* stimuli, which comprised sound-based sculpting with little obvious instrumentation or note-based music, timbre was the primary descriptor. Perceived phrases in response to the instrumental yet primarily sound-based Xenakis *‘Metastaseis’* stimulus were equally categorized by timbre and intensity. However, rhythm was the main descriptor for the single-instrument note-based Beethoven *‘Waldstein’* stimulus (which has relatively narrow timbral range, being the sound of a single instrument). These descriptive results support the study’s hypotheses that phrases can be perceived in environmental sound and sound-based music, and that acoustic factors are recognized by participants. Uncategorizable phrase descriptions (placed in an 'Other' category) were infrequently observed, the exception being for the Beethoven *‘Waldstein’* stimulus, with 51 qualitative responses falling into the ‘Other’ category.

As can be seen in the tables in S1 Appendix, a large proportion of the 51 qualitative responses placed in the ‘Other’ category for the Beethoven *‘Waldstein’* stimulus were responses of ‘no comment’ (39.22%). Nevertheless, we conducted a follow-up analysis investigating whether the inclusion of the ‘Other’ category in models of phrase perception in response to the Beethoven *‘Waldstein’* stimulus altered the main models presented in the Results. Not surprisingly, models were worse when responses from the ‘Other’ category were included in the analysis, relative to the models that only included responses from ‘Intensity’, ‘Timbre’, and ‘Rhythm’ categories; however the effective predictors were hardly changed. We next turn to statistical models designed to determine the relative contributions of intensity, timbre, and rhythm for phrase perception.

### 3.2. Modeling perceived musical phrases: A ‘whole-phrase’ global approach

As indicated, our first modeling purpose was to assess whether the hypothesised influence of intensity, timbre, and rhythmic change on phrase perceptions was plausible and significant (rather than solely to derive the best model of the clustered event processes). Table 2 shows a summary of the models obtained for each piece on the basis of the procedures described above and for all perceived phrases. Table 3 summarizes all predictors used and Table 4 provides the specific predictor coefficients required in each model of the whole-phrase perceptions. The first five stimuli are sound-based (one environmental sound stimulus and four sound-based music stimuli), since they contain few overt rhythmic phrases as judged by our participants (see Fig 1); for these stimuli we do not attempt to define a rhythmic predictor series. The sixth piece, Beethoven’s *‘Waldstein’* sonata contrasts in being highly rhythmic, again as perceived by participants, hence we consider this stimulus to be note-based and we use rhythmic predictor time-series data in the modeling. Pitch (a perceptual feature) is subsumed in the acoustic spectral flatness measure used throughout, but addressed more closely in the later models adding spectral centroid amongst the acoustic timbral predictors. Throughout the remainder of the paper, we discuss results in terms of these sound-based and note-based groupings.

**Table 2:** Summary of selected cox hazard models of perceived phrases using the whole-phrase ‘global’ approach.

Stimulus	*R*^2^	Likelihood Ratio	Model *p-*value	Robust Model *p-*value
BBC SoundFX: *Wind*	.51	67.07	< .001	.002
Ng & Dean: *LowHz*	.42	54.40	< .001	.019
Eno: *Francisco*	.31	37.94	< .001	.003
Wishart: *Red Bird*	.38	69.70	< .001	.004
Beethoven: *Waldstein*	.30	45.32	< .001	.023
Xenakis: *Metastaseis*	.56	82.30	< .001	.060

**Note.** ‘Model *p-*value’ refers to the *p-*value based on the Likelihood Ratio and assumes independence of observations within a cluster (i.e., a group of successive phrase perceptions by an individual listening to a particular piece). The more conservative ‘Robust Model *p*-values’ do not; the time transform used in all these models was *log(t).

**Table 3:** Description of predictors used in cox hazard models of perceived phrases.

Parent Predictor	Description and Potential Range of Values
*M*intens	Mean acoustic intensity (dB SPL)
*M*specf	Mean spectral flatness (Wiener Entropy)
*M*rhythdens	Mean rhythmic density (number of onset events per 500 ms)
*M*spars	Mean sparsity (reciprocal of the number of onset events per 500 ms)
*M*specc	Mean spectral centroid
*M*specsp	Mean spectral spread
*M*specflx	Mean spectral flux
*M*rough	Mean roughness
*M*inharm	Mean inharmonicity
tt(x)	Time transform of the predictor (x)

**Note.** Models included additional versions of each ‘parent’ predictor above: mean change (*M*chg) and mean absolute change (*M*abschg).

**Table 4:** Predictors in the selected cox hazard models of perceived phrases using the ‘whole-phrase’ global approach.

Stimulus	Predictor	Coefficient	Robust *SE* of Coefficient	Coefficient *p-*value
BBC SoundFX	*M*chg specf	34.56	9.49	< .001
	*M*intens	16.32	4.82	.001
	*M*intens:*M*specf	2.93	.89	< .001
	tt(*M*specf)	-15.74	4.82	.001
Ng & Dean	*M*abschg specf	-221.10	83.20	< .01
	*M*intens	-1.36	.26	< .01
	*M*abschg intens	54.60	21.70	.011
	*M*intens:*M*specf *	.02	.02	>.05
	tt(*M*abschg specf)	21.00	7.78	< .01
	tt(*M*abschg intens)	-4.91	2.04	< .05
Wishart	*M*specf	16.61	4.82	< .001
	*M*abschg specf	1.33	.40	< .001
	*M*chg intens	8.83	2.45	< .001
	*M*specf:*M*intens	-.03	.01	.001
	tt(*M*specf)*	-1.03	.53	.051
	tt(*M*intens)	-.71	.01	.001
Eno	*M*specf	-140.30	25.19	< .001
	*M*intens	-20.39	4.46	< .001
	tt(*M*specf)	12.26	2.24	< .001
	tt(*M*intens)	1.79	.39	< .001
Xenakis	*M*specf	-204.90	39.24	< .001
	*M*chg specf	6.10	1.25	< .001
	*M*intens	-19.84	2.16	< .001
	*M*chg intens	.51	.10	< .001
	*M*abschg intens	-.93	.16	< .001
	*M*specf:*M*intens	-.12	.02	< .001
	*M*chg specf:*M*chg intens	-1.55	.32	< .001
	tt(*M*specf)	18.48	3.50	< .001
	tt(*M*intens)	1.62	.18	< .001
Beethoven	*M*chg rhythdens	1.97	.68	< .01
	*M*abschg rhythdens	44.10	8.60	< .001
	*M*spars	5.61	1.66	< .001
	*M*chg spars	11.43	2.61	< .001
	*M*asbchg spars	-8.12	2.81	< .01
	*M*specf:*M*rhythdens	-.47	.22	< .05
	tt(*M*abschg rhythdens)	-.06	.02	< .01

**Note.** Asterisked (*) predictors are individually non-significant but nevertheless required in the selected model. A colon placed between the two predictors denotes an interaction between them; *p-*values rounded to three decimal places. The time transform used in all these models was *log(t).

For sound-based items, selected models included acoustic predictors based on intensity, spectral flatness, and their interaction (with the sole exception of Eno’s ‘*Francisco’*, where no interaction was observed). These results suggest that both intensity and spectral flatness are perceptually important in listeners' processes of identifying musical phrases. Furthermore, the descriptions and model interactions indicate that listeners vary in the degree to which these two acoustic features are considered important at any moment, and they may well conflate the two at times. The selected models included logarithmic time-dependency components (as discussed in methods; abbreviated ‘tt’ in Results Tables) for intensity and spectral flatness.

As expected, the note-based Beethoven *‘Waldstein’* stimulus was modeled in different ways to the other five sound-based stimuli. The tempo of the piece is mostly 120 crotchets (1/4 notes) per minute; therefore a ‘rest’ of one crotchet might occupy one 500 ms window (which would then have a rhythm count of zero). As can be seen in Table 4, spectral flatness but not intensity was required for the selected model, and parameters specific to the rhythmic structure of the piece were dominant (seemingly replacing the intensity profile), even though the overall model fit was still relatively poor (*R*^2^ = .30). When listening to the Beethoven *‘Waldstein’* stimulus, one can identify intermittent gaps in the activity of the music, where the generally ongoing quaver or semiquaver (i.e., 1/8 or 1/16 notes) note sequences rest, often at points of cadence. These are analogous to the pauses between spoken sentences or turn-taking between multiple speakers in conversation [59]. The role of rhythm in the Beethoven model thus supports the decision to instruct listeners to consider such analogies when completing the experiment.

Given that most models showed both intensity and spectral flatness to be significant predictors, we undertook some models where the series of perceived phrases categorised by intensity and timbre were considered separately. Two stimuli were chosen for intensity and timbre: BBC ‘*Wind’* (comprising the largest number of intensity phrases) and Wishart’s *‘Red Bird’* (comprising the largest number of timbre phrases). A corresponding analysis of rhythmic phrases for the only relevant case, the Beethoven ‘*Waldstein’* stimulus, was also made. Suffice to say that the optimum models were not improved or changed in form when categories of phrase responses were analysed as separate streams rather than one continuous stream.

Since the Beethoven *‘Waldstein’* model was rather poor, and intensity was not a predictor, we assessed whether rhythmic gaps might have a special role in perception of its phrases. A binary variable, where a gap is coded 1, and ongoing rhythmic events 0, was too impoverished a representation to contribute to the model (there were only 14 such gaps, concentrated in four regions in the context of a total of 292 x 500 ms windows). As a result, we developed a 'sparsity' measure that was the reciprocal of the number of note or chord onsets per 500 ms sample. All sparsity values derived this way for samples containing events were between 0–1, and we arbitrarily re-coded gaps (i.e., time slices with no events) as 2 (instead of infinity). Table 4 shows that this sparsity measure was a significant predictor in our model of the Beethoven *‘Waldstein’* stimulus, alongside the previous parameters specific to the rhythmic structure. This suggests that as hypothesized, perceptions of sparsity ('pauses' or gaps in activity) may have an impact distinct from perceptions of rhythmic density. These models are intuitively comprehensible and may indicate a form of 'regime switching' (or thresholding) whereby the sparsity component is important in the highly sparse parts, but not otherwise. However, this model goes against several principles of parsimony; for example, it uses a data stream twice (counts of absolute rhythm). The complete model had an *R*^2^ value of .30; without the sparsity measure the *R*^2^ value was .18; without absolute counts of rhythmic events, the *R*^2^ value was .06. Thus, both rhythmic density and sparsity contributed to hazard modeling (i.e., the likelihood of a perceptual event) of the Beethoven *‘Waldstein’* stimulus. Unsuccessful attempts were made to use the log or CoxBox transform to unify the density and sparsity measures into a single predictor.

### 3.3. Impact of terminal acoustic features on perceived musical phrases

In further pre-planned analyses, we replaced the 'global' acoustic data obtained from the whole of each phrase with data obtained only over its last five seconds; the ‘terminal’ portion of each perceived phrase. This tested whether the acoustic factors within the terminal portion play particularly important roles for phrase perception, above and beyond the acoustic information across the complete duration of perceived phrases. We hypothesized at the outset that the terminal portion of each phrase would have a relatively strong impact on phrase perception when phrase durations extend beyond five seconds. This temporal window was chosen on the basis of prior evidence from time series models of several continuously perceived aspects of pieces such as those presented herein (notably, perceived musical ‘change’ and affective arousal and valence) [16, 19–22, 26–28, 54]. In such models lags of predictors up to five seconds in duration and beyond are required for optimal models. Furthermore, research implementing Markov chain models of musical structure and perception report similar results [60]. Thus we assessed whether using the same acoustic predictors measured solely over the last five seconds of each perceived phrase could result in models that were as good or even better than those obtained from the whole-phrase trajectory. Given that the mean duration of perceived phrases was 15.05 seconds across all participants and pieces, the terminal five seconds of each phrase corresponds to roughly the final third.

Results from this analysis confirm that a combination of intensity and spectral flatness predictors, together with time dependence, remained in each model of the sound-based stimuli. The precise form of the models changed somewhat, but given the retention of the acoustic predictors, we compare *R*^2^ values in Table 5 between the selected models that include acoustic information over each phrase’s entirety (the ‘global’ approach) with the selected models that include acoustic information solely from the final five-seconds of each phrase (the ‘terminal’ approach). As can be seen in Table 5, in half the cases acoustic information over the final five seconds of a phrase provided a model that was predictively slightly better than those using the whole-phrase acoustic data. In the other half, the models were slightly inferior. As above for global models of the Beethoven stimulus, rhythm and sparsity predictors remained significant for its 'terminal' model, together with spectral flatness. The results suggest that the terminal portion may sometimes have particular influence, but improvements due to the terminal portion of acoustic information were not uniform.

**Table 5:** Comparison of *R*^2^ values between whole-phrase ‘global’ models and ‘terminal portion’ models.

Stimulus	Whole-Phrase ‘Global’ Models	‘Terminal Portion’ Models
BBC SoundFX: *Wind*	.51	.52
Ng & Dean: *LowHz*	.42	.52
Eno: *Francisco*	.31	.28
Wishart: *Red Bird*	.38	.42
Beethoven: *Waldstein*	.30	.28
Xenakis: *Metastaseis*	.56	.47

### 3.4. Models including additional spectral parameters

As described in the Methods section above, a rational selection of spectral parameters was measured to supplement the routinely used spectral flatness, as potential predictors of perceived timbre and its impact on phrase perception. These were spectral centroid, flux and spread, together with inharmonicity and roughness. A standard procedure (see Method section) was used to select an improved parsimonious model. Table 6 summarizes the results and Table 7 shows the specific predictor coefficients in each model. Overall, *R*^2^ values were improved noticeably for all five sound-based stimuli; the improvement for the note-based piece was trivial. Spectral flatness and intensity were retained within the models of the five sound-based stimuli; spectral flatness and rhythmic density were retained in that of the note-based ‘*Waldstein*’ Sonata (intensity was not included in the earlier model either).

**Table 6:** Summary of selected cox hazard models of perceived phrases using the whole-phrase global approach and additional spectral parameters.

Stimulus	*R*^2^	Likelihood Ratio	Model *p-*value	Robust Model *p-*value
BBC SoundFX: *Wind*	.67	103.20	< .001	.030
Ng & Dean: *LowHz*	.57	84.15	< .001	.103
Eno: *Francisco*	.50	72.12	< .001	.013
Wishart: *Red Bird*	.60	134.00	< .001	.032
Beethoven: *Waldstein*	.32	48.32	< .001	.021
Xenakis: *Metastaseis*	.64	130.00	< .001	1.000

**Note.** ‘Model *p-*value’ refers to the *p-*value based on the Likelihood Ratio and assumes independence of observations within a cluster (i.e., a group of successive phrase perceptions by an individual listening to a particular piece). The more conservative ‘Robust Model *p*-values’ do not; the time transform used in all these models was *log(t).

**Table 7:** Predictors in the selected cox hazard models of perceived phrases using the global approach and additional spectral parameters.

Stimulus	Predictor	Coefficient	Robust *SE* of Coefficient	Coefficient *p-*value
BBC SoundFX	*M*intens	4.32	.62	< .001
	*M*abschg specc	-.06	.01	< .001
	*M*specsp	-.02	.01	< .001
	*M*abschg specflx	349.70	41.88	< .001
	*M*rough	2956.00	433.10	< .001
	*M*inharm	-47.47	17.73	< .01
	*M*abschg inharm	101.80	29.02	< .001
	*M*intens:*M*specf	.90	.11	< .001
	tt(*M*specf)	-.01	.01	< .001
Ng & Dean	*M*intens	-11.57	3.56	< .01
	*M*specf	216.00	72.95	< .01
	*M*abschg intens	3.47	.78	< .001
	*M*specc	.01	.01	< .01
	*M*specsp	-.01	.01	< .001
	*M*chg rough*	-269.40	154.60	>.05
	*M*inharm	44.74	13.50	< .001
	*M*chg inharm	-35.11	15.02	< .05
	*M*intens:*M*specf	-2.99	1.03	< .01
	tt(*M*abschg intens)	-.01	.01	< .01
Wishart	*M*specf	72.37	18.12	< .001
	*M*specc	-.03	.01	< .001
	*M*chg specc	.02	.01	< .001
	*M*abschg specc	.02	.01	< .01
	*M*rough	332.00	58.50	< .001
	*M*inharm	63.69	18.58	< .001
	*M*chg inharm	-149.50	29.11	< .001
	*M*abschg inharm	78.96	25.11	< .01
	*M*specf:*M*intens	-.07	.02	< .001
	tt(*M*specf)	-5.52	1.40	< .001
Eno	*M*intens	-25.05	7.62	< .01
	*M*specf	-161.40	37.13	< .001
	*M*specc	.02	.01	< .001
	*M*chg specflx	-24.91	8.25	< .01
	*M*rough	-1893.00	370.20	< .001
	*M*chg rough	-1827.00	401.00	< .001
	tt(*M*specf)	13.99	3.32	< .001
	tt(*M*intens)	2.17	.68	< .01
Xenakis	*M*intens	-3.35	.36	< .001
	*M*chg intens	.21	.07	< .01
	*M*abschg intens	-1.02	.13	< .001
	*M*chg specc	.02	.01	< .001
	*M*specsp	-.03	.01	< .001
	*M*intens:*M*specf	-.16	.02	< .001
	*M*chg intens: *M*chg specf	-1.13	.25	< .001
	tt(mspecsp)	.01	.01	< .001
	tt(mintens)	.01	.01	< .001
Beethoven	*M*abschg rhythdens	37.89	7.23	< .001
	*M*spars	5.28	1.23	< .001
	*M*chg spars	6.92	1.92	< .001
	*M*abschg spars	-7.33	1.57	< .001
	*M*specsp	-.01	.01	< .05
	*M*inharm	-28.15	2.09	< .001
	*M*specf:*M*rhythdens	-.05	.02	< .05
	tt(*M*abschg rhythdens)	-3.21	.63	< .001

**Note.** Asterisked (*) predictors are individually non significant but nevertheless required in the selected model. A colon placed between the two predictors denotes an interaction between them; *p-*values rounded to three decimal places. The time transform used in all these models was *log(t).

The selected model for the note-based *Waldstein* sonata also included modest contributions from spectral spread and inharmonicity (but not from spectral centroid). Spectral centroid is not equivalent to pitch, even for a monophonic sound (in that case, centroid is almost always significantly higher than pitch or fundamental frequency, because of higher harmonic and inharmonic partials), unless pitch is above about 4000Hz. With monophonic sounds, spectral centroid commonly changes in the same direction as pitch, but with polyphonic note-based sounds it more closely approximates the average pitch of the component tones. The lack of predictive power of spectral centroid in the Beethoven *‘Waldstein’* models thus suggests that the mean pitch was not a major influence. In contrast, the pitch range is more clearly represented in the acoustic spectral spread values, which were a predictor for the Beethoven *‘Waldstein’* stimulus, suggesting that pitch range (which varies dramatically) was probably important here for phrase perception. For the sound-based stimuli, spectral centroid (all five stimuli), spectral flux (*‘BBC Wind’* and *‘Francisco’*), spectral spread (*‘BBC Wind’*, ‘*Metastaseis’*, and *‘LowHz’*), inharmonicity (*‘BBC Wind’*, *‘LowHz’*, and *‘Red Bird’*) and roughness (*‘BBC Wind’*, *‘Francisco’*, *‘LowHz’*, and *‘Red Bird’*) were additional contributors to the models.

## 4. Discussion

Our mixed methods approach investigated the occurrence and nature of listeners’ perception of musical phrases in sound-based musical stimuli that comprise few of the pitch-related events most commonly found in instrumental note-based music. The range of stimuli also included note-based instrumental music and environmental sound. Fig 1 shows that phrase perception was achieved in a coherent manner (i.e., the expected clustered recurrent event process). The qualitative results in Table 1 and the tables in S1 Appendix clearly show that for the four stimuli we categorized as ‘sound-based music’, listeners described phrase features most frequently as aspects of timbre (331 phrases in total) and less frequently as aspects of loudness, which we label 'Intensity' as our operational acoustic measure (104 phrases in total). Rhythm was not important for perceived phrasing in these stimuli. The sound-based musical excerpt that involved orchestral instruments (Xenakis’s *‘Metastaseis’*) was perceived as comprising slightly more intensity-related than timbre-related phrases, suggesting that instrumental activity and note-like attacks in the piece were perceived more readily here than in the other three. Close listening to all four sound-based music stimuli supports this view. The fifth sound-based stimulus, the environmental (rather than musically composed) sample of a BBC field recording of wind noises, was predominantly perceived in terms of intensity-related phrases (68), with only 24 cases associated primarily with timbre. The fact that intensity was relatively predominant for the Xenakis ‘*Metastaseis’* and BBC ‘*Wind’* stimuli is again consistent with the overall characteristics of each stimulus, as timbral change in both examples is modest. Indeed, examples of major timbral change are sparse in the Xenakis ‘*Metastaseis’* stimulus, even though the music is performed using a wide range of orchestral instruments. When timbral change is apparent in ‘*Metastaseis’*, brass and percussion events tend to supervene over the strings. Such changes of instrumentation were noted in at least 30 of the descriptions shown in Table E in S1 Appendix, and in several cases, both loudness and instrumental changes were mentioned simultaneously. Furthermore, some participants displayed awareness of several individual brass, percussion, and string instruments. At least 11 descriptions referred to an abrupt silence in this extract, where sound drops out briefly: they used words such as 'stop' and 'gone', clearly indicating a cessation of activity (or a 'gap'). A prior detailed analysis of acoustics and affective responses to *‘Metastaseis’* [61] is consistent with these observations, including the role of silence (which was therefore not investigated further in the present work).

Even more than the Xenakis ‘*Metastaseis’* stimulus, the BBC ‘*Wind’* stimulus is relatively homogeneous in character (being environmental rather than compositional) and thus, timbre did not play a significant role in determining listeners’ perception of phrasing. The fact that environmental sounds can be systematically segmented is to be expected from the evolutionary considerations of ecological acoustics [62–64]. One may speculate that most human auditory segmentation is based on abilities required biologically and subsequently molded by experience of speech. The fact that the average duration of perceived phrases in our study was around 15 seconds, and much greater than the minimum implied by our task demands (~5 seconds), confirms that our listeners were normally dealing with aggregations of short events. There is evidence that word duration is related to information content [65], and this may apply also to longer units such as linguistic clauses, sentences and musical phrases, and this relationship may be reflected in the magnitude of change in acoustic parameters.

As hypothesized, the instrumental note-based Beethoven *‘Waldstein’* stimulus resulted in dramatically different data when compared to the five sound-based stimuli. Notably, phrases where rhythmic events were described as the key attribute (64) were observed at a greater frequency than those categorized by either timbre (32) or intensity (31). Similarly, while rhythmic phrases comprised more than 50% of those perceived in the Beethoven stimulus, they comprised less than 7% of phrases in any of the sound-based stimuli.

### 4.1. Models of phrase perception

The qualitative descriptors supported our intention of modeling the predictive influence of acoustic features upon perception of phrases. We started with simple models involving only single, 'global' measures of timbre (spectral flatness, a feature at the top of the MPEG-7 descriptor tree), intensity, and rhythm, as chosen in our earlier work. The measures were taken with three different values to represent the behavior of each acoustic parameter across the whole duration of each perceived phrase. The three values represented the mean, the mean change, and the mean absolute change in each parameter in the specified window, as previously recommended [53] and useful in other contexts [66]. Reasonable models were obtained using Cox recurrent event hazard analysis, with *R*^2^ values ranging between .30 and .56. For sound-based stimuli, optimum models required contributions from spectral flatness, intensity, and for all but one (Eno’s ‘*Francisco*’), their interaction, confirming the importance of both acoustic parameters and their likely perceptual interaction in the determination of phrases. There were clear signs of temporal variation across responses to each sound-based extract, consistent with the idea that these stimuli were unfamiliar, with responses evolving as exposure increased.

As hypothesized, the Beethoven *‘Waldstein’* note-based stimulus was modeled very differently, with intensity a redundant predictor; in a sense it was replaced by the measure of rhythmic density and its interaction with spectral flatness. This interaction was the only component by which spectral flatness contributed in models of Beethoven’s *‘Waldstein’*, confirming its subordinate role here. Similarly, the coefficient for the time dependence of the absolute change in rhythmic density series was here much smaller than any such coefficients in the sound-based stimuli. This observation is consistent with the greater familiarity of note-based music. Pauses in rhythmic activity, reflected in our sparsity measure, had quite a strong role that was separable from that of rhythmic density, and somewhat like the role of the major ‘gaps’ in the Xenakis *‘Metastaseis’* orchestral stimulus [67]. Future models of such music may establish a more parsimonious way of modeling both rhythm and sparsity that derive from one measured series only, rather than modeling the two parameters we derived here. It was not our purpose to broach this further, but rather, to focus on timbral aspects instead.

Our models of the isolated intensity, timbre, or rhythm phrase series from the three stimuli in which these features were most important were consistent with the 'whole-phrase' series models. Notably, in separate models of the timbral and of the intensity series, both spectral flatness and intensity remained mutually required predictors, confirming the above observations of their ongoing interaction. In addition, it is quite likely that listeners may have sometimes confused them in their descriptors, just as the tables in S1 Appendix show that sometimes both timbre and intensity were included in overlapping phrase descriptions by different participants.

We hypothesized that the acoustic features of the final portion of a perceived phrase might be the dominant influence on its perception. As Table 5 shows, we were able to obtain models using only the data of the last five seconds of each phrase that were comparable to (but not uniformly better than) those that used the whole-phrase data. The models were similar in the range and relative coefficients of the predictors involved, and so the last five seconds were sufficient but not 'dominant'. This may be due to the fact that the phrases were relatively homogeneous, so that the measured parameters of the last five seconds were similar to those of the whole phrase. We return to the practical applications of this observation below.

### 4.2. Analyzing the role of several spectral descriptors

As indicated in the Introduction, we did not assume that models of dissimilarity between short separated individual sounds (the main form of timbral characterization studied to date) would be immediately applicable to the circumstances of sound-based music, where sonic continuities predominate over separations. Even the modest disparities between key predictors of instrumental sound dissimilarity models [53] and those of environmental sounds [68, 69] support the view that some differences are to be expected with our longer continuous sounds, as do the few earlier works on perceived segments in continuous sound pieces described in the Introduction [26–28]. We chose a representative selection of spectral descriptors on the basis of their previous utility and relatively low correlation in models of instrumental note-based sound, and applied them to our whole-phrase perception data.

Consistent with their important positions in the MPEG-7 descriptor system, both intensity and spectral flatness were retained within the resultant more complex models of all sound-based extracts, which were all improvements over the simpler models. They were joined variously by spectral centroid, inharmonicity, roughness, spectral spread, and less commonly, spectral flux. This last observation was perhaps the most interesting, since spectral flux has been reported as a significant factor in models of dissimilarity of isolated pairs of sounds, such as those created by musical instruments [53], but not environmental sounds [68, 69]. A reasonable interpretation is that spectral flux is relatively high throughout these sound-based extracts (and in natural environmental sounds beyond our example), somewhat as it is in speech. The Beethoven *‘Waldstein’* (note-based) model was not substantially improved in fit by the additional choice of spectral predictors, but did introduce spectral spread and inharmonicity. This result presumably reflects the variation in keyboard ranges over which the extract operates, and the fact that the piano is uniquely characterized by its inharmonic components. Thus again, this note-based piece revealed influences of spectral components in addition to rhythm. We conclude that the selected range of added spectral predictors were broadly appropriate because all made contributions in some cases. Therefore, they are suitable for use in future studies of sound-based music.

One limitation of our study is that we had no way of separating the spectral fluxes in different simultaneous streams of the music. While our models were moderately good, they might well be improved were such precise computational stream segregation readily feasible, as it no doubt will be in the near future. However, our concern was with ecologically valid musical (and environmental) extracts, and it proved adequately successful.

### 4.3. Relations between perceptual segmentation of speech and music

The conclusions just discussed are consistent with those from studies of perception of speech clauses and sentences, the analogue of musical phrases. This suggests that the categorization of languages into stress- and tone-based may have a deeper relation with that of note- and sound-based music than just analogy. This possibility has not been noted previously [70], presumably because of the paucity of studies on sound-based music. For example, the relationship may suggest that rhythmic events can be delineated by the recurrence of purely timbral changes (even without intensity changes), and listeners can progressively learn to identify them. More specifically, those timbral changes may not require either pitch change or change in spectral centroid (perhaps a more nuanced single dimension representation of the multidimensional frequency spectrum of a sound than pitch). However, we should not speculate too far on this possible relationship in the absence of relevant empirical data.

Returning to the broader relation between speech and music perception, we note that ‘pauses’ delineate speech clauses and sentences, and phrases in sound-based music, not only when there is a gap with a virtual absence of sound, but also when the pause is constituted by a more subtle slowing of change of particular spectral parameters. Perceptual measures of continuous change in music from which intensity change has been removed will be informative in this respect, and these can be coupled with studies of perceived phrase segmentation in the same conditions.

The motor theory of speech cognition has long held that auditory and visual cues to speech comprehension are accompanied by a motor appraisal, in which the physical processes necessary for generating the observed sound are assessed by the listener and subsequently contribute to the ultimate cognitive categorization of the speech sound and its intelligibility [71]. Extensive criticism of this theory exists, partly on the grounds of unnecessary complexity [72]. The theory bears consideration in relation to music in light of Godøy's theories on chunking of music through co-articulation [73, 74]. Here, a chunk is the fusion of brief physical generative actions and sounds into larger ones. These superordinate units are considered to have a mean duration of about three seconds (shorter than most we encouraged and observed in the present study) and to result from either 'exogenous' or 'endogenous' factors. Exogenous factors, considered consequential on physical actions of a sound-producing performer, provide clear 'discontinuities' in the signal and induce the perception of start and end points. This points to instrumental notes and the correlations between performer movement and note onsets and offsets; features largely absent in all our extracts except the note-based Beethoven *‘Waldstein’* piano stimulus (it was for this reason that we presented the two instrumental works, Beethoven and Xenakis, at the end of our stimulus presentation sequence). Endogenous factors are 'top-down' projections by the perceiver that result in perceptions that are less consistent between individuals. While co-articulation for Godøy is the co-temporality of the performer's actions and musical chunks, he also notes the use of the same term in speech perception to indicate the smearing of phonemic sound components and in some cases, their alteration, which derives from the succession of physical movements required to generate a particular sequence of phonemes.

The so-called 'resonant' perception of speech components [75] involves integration of chunks, including backwards integration in time. In other words, aspects of chunks are kept in memory, sometimes beyond working memory, allowing holistic perception. In music [76], this might be the 'temporal anamorphosis' proposed by influential sound-based composer-theoretician Pierre Schaeffer [77] and corresponds well with the phrases we describe in this paper. While the phrases in most sound-based music do not derive from physical aspects of performance, they may be constructed compositionally so that they sound analogous to such performance, perhaps because of the connections we have raised with speech or those with larger evolutionary and environmental acoustic factors. Our evidence for the role of perceived agency in perception of affect in sound-based music suggests this connection may be present but modest [78].

### 4.4. Phrases in note-based music

There is a large literature on segmentation of note-based, usually monophonic (single stream) proto-musical experimental sequences. A suitable basis for considering this in our present context is the recent in depth introduction and study by Hutchison et al. [79]. These authors went beyond proto-stimuli to include excerpts of a compilation of folk songs, although most were still monophonic. The study primarily compared the predictive accuracy of the perceived phrase structure model (PPS), and the generative structural grammar model (GSGM) of segmentation; that is, the degree to which they could predict users' perceptions of segmentation. It also considered an information theoretic model, the information dynamics of music (IDyOM). Interestingly, only marginal effects of participants’ musical expertise were observed on model prediction accuracies. This suggests that with note-based music either there is no effect of degree of familiarity, or participants had reached a ceiling for such effects. We consider the latter a more likely interpretation.

As formulated, these models are almost entirely focused on 'notes', though the GSGM model does involve one grouping rule that considers five categories of 'change in the music' that include dynamics (loudness) and timbre/instrumentation. Thus the models are largely inapplicable to sound-based music in their initial formulation, although they (and most readily IDyOM) could be rephrased in terms of other musical parameters (such as an MFCC description of a series of timbral events). One notable feature emphasized directly in the PPS model and applicable to sound-based music and our results, is its Rule 1: 'Gap'. PPS suggests that large inter-onset intervals and most importantly, large offset-to-onset intervals tend to result in phrase boundary perception. This is entirely consistent with the speech literature and the observations and interpretations in the present paper. IDyOM's analysis also takes account of such gaps.

### 4.5. Potential implications

The speech perception literature has at least one more implication. How does statistical learning of relationships between segments proceed? Learned words can function as 'anchors' facilitating the learning of others [80]. More importantly, if the start and finish of a linguistic phrase is identified (on the basis of the ‘bootstrapping’ processes discussed above), then it may be possible for a learner to pick off units that then help define others. Johnson [40] gives the following simple example: an English language learner hears ‘Look. Look here. Here is the cat.’ ‘Look’, presented in isolation, is identified as a word. Then it can be subtracted from 'Look here', revealing that 'here' is probably a word too. The process can continue according to the language elements presented.

Our interpretations have application to additional ‘real-world’ contexts in which the formation of cognized musical phrases occur. The first application is to provide data informing electroacoustic and algorithmic music composers who commonly implement spectral transformations by DSP techniques that are salient for perception of musical phrases in sound-based music. This is important because ultimately, sequences of phrases (and recognition of phrase similarities) are the elements of music that contribute to its structure and affective qualities. It is perhaps not surprising (but nevertheless useful) to realize that even in continuous sound environments, spectral flatness and spectral centroid are both important (as found earlier with a sound-based piece; see [20]), together with inharmonicity, roughness, and spectral spread. The relative lack of influence from spectral flux in the present study does not of course rule out its importance, but rather suggests that it is a descriptor whose impact may be driven more directly by component factors such as changes in the individual spectral factors discussed above. This interpretation seems consistent with data using synthetic tones [81]. Furthermore, the powerful role of temporal changes in acoustic intensity for music perception (e.g., subjective and physiological affective responses [22, 54, 82]) is also well supported by the present data.

The second application of the work is specific to the question: how might one best consider timbre and its relation to affect in the context of music information retrieval (MIR) approaches to music recommender systems? Broadly, MIR is the large discipline in which computational information about music (such as acoustic or structural information) is used to identify similarities, relationships, and even genres, largely using analytical and machine learning techniques [34]. Music recommender systems apply such information together with social use and preference data taken with demographics and individual use histories, primarily to recommend new music to listen to or purchase (such as with Amazon, iTunes, Spotify, or Shazam). We are seeking in other work to extend 'affective'-based music recommender systems (which recommend pieces of music that bear affective similarity to other pieces experienced and liked by a user) by exploiting information from short segments of listeners’ continuous affective responses and apply it to the exploration of unfamiliar music libraries (rather than commercial libraries driven by promotion and social usage).

To this end, we require a process that is able to quickly and accurately model the features of musical timbre that influence perceptions of a particular user, especially in the context of the affective profile from a segment of music they have heard. We envisage that modeling such data on the basis of temporal data chunks of sufficient duration to predicate phrase perception may at least be competitive with current recommender approaches that simply implement a version of the ‘bag of frames’ approach (i.e., to use short pieces of acoustic information with no particular known relevance to perceived affect). Using chunks such as the five second windows indicated in the present study may allow more accurate and faster constructed individual-centered models that can be used in future personalized music recommender systems. In our future work, we plan to make such comparisons.

In conclusion, this study has demonstrated the occurrence, relevance, coherence and distinctiveness of perceptions of musical phrasing in sound-based music. It shows clear roles of acoustic intensity and a range of timbral features therein. The results will form the basis of future empirical studies designed with applications to contexts such as electroacoustic composition and music recommender systems.

## Supporting Information

S1 Data Complete dataset. (XLSX)

S1 Appendix Qualitative descriptions and designated categories of perceived phrase responses. (PDF)
