# Supplementary material for: What Constitutes a Phrase in Sound-Based Music? A Mixed-Methods Investigation of Perception and Acoustics
Source: PLoS One. 2016 Dec 20;11(12):e0167643. doi: 10.1371/journal.pone.0167643 (PMC5172564; doi:10.1371/journal.pone.0167643)
Supplement: S1 Appendix — (PDF) [file pone.0167643.s002.pdf]

Supporting Information 1: Appendix

Table A. Qualitative descriptions and designated categories of perceived phrase responses to BBC SoundFX (1989) 'Tree Creaking in Strong Wind'

Qualitative Description Explaining Response	Category
Sound intensified	Intensity
Louder, closer	Intensity
Quieter	Intensity
Loud	Intensity
Sounds soften	Intensity
Quieter, creaking louder	Intensity
Softer	Intensity
The waves died down for a second	Intensity
Became quieter and a visible creaking sound began	Intensity
Waves have dropped in volume	Intensity
Getting louder, new wave	Intensity
Loud, soft and back to loud	Intensity
The waves died down for a second	Intensity
Sound softened, creaking	Intensity
Sounds soften	Intensity
Lower sound	Intensity
Waves drop and a stretching sound is becoming louder	Intensity
Louder	Intensity
Soft to really loud	Intensity
Becomes aggressive	Intensity
Sound intensified	Intensity
Sound gets deeper	Intensity
Sounds of trees blowing harder	Intensity
Heavier	Intensity
The rain subsided slightly, I felt it was calmer	Intensity
Getting louder again	Intensity
Calm, soft and just a little loud towards the end	Intensity
The rain was consistent then changed into more random levels of intensity	Intensity
Sound increased	Intensity
Rains harder	Intensity
Loud, dramatic	Intensity
Rain is getting heavier	Intensity
Waves are loud and overpowering	Intensity
Getting louder and more intense	Intensity
Heavier noise	Intensity
Quieter, anticipating it getting louder	Intensity
Loud but from a distance	Intensity
Wind is picking up and the chair is rocking more violently	Intensity
Creaking noise is back seems louder now	Intensity
Rain softer	Intensity
Loud to soft and calm	Intensity
Sound decrease	Intensity
Waves have gone down in volume, the scratching sound is back	Intensity

Sound gets lighter	Intensity
Soft and build up to loud	Intensity
Rains harder	Intensity
Sound intensified	Intensity
Getting louder	Intensity
Everything is louder	Intensity
Louder	Intensity
The wind has become stronger	Intensity
Loud, dramatic, closer	Intensity
Heavier noise	Intensity
Sound increase	Intensity
Getting quite aggressive, worried	Intensity
The sound increased and sounded different. Like the rain became heavier, violent	Intensity
Sound intensified	Intensity
Waves started to get really loud	Intensity
Rain seems heavier more intense	Intensity
The sound of waves in background	Intensity
Rains harder	Intensity
Gets deeper	Intensity
Sustained increase in storm intensity	Intensity
Increased sound and creaking has disappeared	Intensity
Very loud, close	Intensity
Quiet	Intensity
The sounds are still there but it is softer	Intensity
From loud to soft	Intensity
Creak slow and drawn out	Rhythm
Rain begins to slow down	Rhythm
Higher wind	Timbre
Heavy rain	Timbre
Creaking	Timbre
Creaking	Timbre
Rain got lighter	Timbre
Creaking of the chair came back	Timbre
Creaking	Timbre
There was more focus on the creaking sound	Timbre
There was a scraping noise	Timbre
Creaking more consistent	Timbre
Although the same event is happening a new sound is introduced	Timbre
Swooshing of the waves are almost non existent	Timbre
Could hear the scraping noise more clear	Timbre
Wind peaking	Timbre
Windy	Timbre
New wave	Timbre
New sound introduced very subtle though	Timbre
Creaking	Timbre
There was a different sound introduced	Timbre
Rain is picking up and chair is rocking	Timbre
Creaking began	Timbre
Sound decrease	Timbre
Another wave	Timbre
Small waves breaking	Timbre

Stayed the same	Other
Becomes normal	Other
Stayed the same	Other
Stayed the same	Other

Table B. Qualitative descriptions and designated categories of perceived phrase responses to Ng and Dean (2000) 'LowHz'

Qualitative Description Explaining Response	Category
Very loud and instrument changed	Intensity
Louder and different use of instruments	Intensity
Very loud	Intensity
Static	Intensity
Very loud, different instruments	Intensity
Really loud	Intensity
Static	Intensity
Loud and change of instrument	Intensity
Very loud	Intensity
Loud	Intensity
Loud, instruments would change	Intensity
Louder, going in and out of instruments	Intensity
Getting very loud and change of tone, noise and instruments	Intensity
Loud, going in and out of a different instrument	Intensity
Very loud and developing more noise and getting more louder	Intensity
Getting louder	Intensity
Louder, change of instrument	Intensity
Helicopter getting louder	Intensity
Louder, a lot more background noise and different instruments	Intensity
Getting louder	Intensity
Getting louder, more background noise	Intensity
Very loud, a lot of background noise	Intensity
Louder, background noise	Intensity
Loud, different use of background noise	Intensity
Very loud and background noise	Intensity
Faster	Rhythm
Rhythm changed	Rhythm
Increased speed and introduction of new sounds	Rhythm
That specific sound follows a regular beat	Rhythm
It sounds faster	Rhythm
The alarm-like sound changed rhythm with an added beat	Rhythm
The sound follows a regular pace	Rhythm
Noise became halting	Timbre
Drumming stopped	Timbre
Buzzing is back	Timbre
Screech	Timbre
Change of tone	Timbre
New alien sound added	Timbre
New instruments	Timbre
Sounds like a new instrument is being added	Timbre

A new level of static sounds was introduced, more instrumental	Timbre
New base sound	Timbre
Changed/extra noise came in	Timbre
Whirring noise started	Timbre
New sound introduced	Timbre
Added distortion over the base rhythmic event	Timbre
Electric guitar?	Timbre
Tone of music changed	Timbre
More white noise	Timbre
Noise became smooth	Timbre
Another level of sound was introduced of a higher pitch	Timbre
Additional instruments used	Timbre
New sounds	Timbre
Distorted growling sound begins	Timbre
Rain	Timbre
Another sound added flashing sound	Timbre
Change of tone	Timbre
Change in sound	Timbre
Alien talking sound	Timbre
Screeching	Timbre
Noise became halting	Timbre
Scratch sound	Timbre
Screeching	Timbre
Change in screeching	Timbre
Helicopters	Timbre
A helicopter has arrived!	Timbre
Helicopter noise began	Timbre
Sounds like a helicopter in the background	Timbre
One of the sounds changed to become more consistent	Timbre
Building up of instruments and getting louder	Timbre
Helicopter in background?	Timbre
Helicopter blade sound starts	Timbre
More rumbles	Timbre
Screeching nearly gone	Timbre
Everything seems deeper	Timbre
Helicopter coming	Timbre
Helicopter more noticeable	Timbre
Sounds like a helicopter in the background	Timbre
New sound	Timbre
Propeller sound prominent	Timbre
More instruments and getting louder	Timbre
Sound of a tracker interferes	Timbre
A new noise, loud	Timbre
Electric guitar has stopped	Timbre
More deep sounds	Timbre
New deep sound	Timbre
More focus was placed on an alarm-like sound	Timbre
Alarm noise began	Timbre
Lower pitch alarm noises in background	Timbre
Original sound at the beginning has disappeared, a loud drilling sound has joined	Timbre
Didgeridoo sound	Timbre

Siren sound prominent	Timbre
New sound was introduced	Timbre
Subtle scream	Timbre
Helicopter close	Timbre
Sounded like a telephone was ringing	Timbre
Repeating sound in background	Timbre
Scratching of metal	Timbre
Developing a new instrument	Timbre
Helicopter back	Timbre
Stayed the same	Other
(No comment)	Other
(No comment)	Other
Sounds closer	Other
(No comment)	Other

Table C. Qualitative descriptions and designated categories of perceived phrase responses to Wishart (1977) 'Red Bird, a political prisoner's dream'

Qualitative Description Explaining Response	Category
Background music was a lot louder	Intensity
Background noise loud	Intensity
Softer	Intensity
Sound decreased	Intensity
Music went softer	Intensity
Everything is getting quite	Intensity
Quieter	Intensity
Louder hit sound	Intensity
The background noise is getting louder and more dominant	Intensity
Pause in sound	Intensity
The sound was louder	Intensity
Banging faster and louder	Intensity
The fly seems to be killed with the loud bang, also sounds like the last bang as it slightly echoed	Intensity
The buzzing sound and book sounds stopped with a conclusive tone	Intensity
Dropping sound getting lower	Intensity
Louder and closer	Intensity
Door closing is louder and shifted in sound to create a rhythm	Intensity
Loud noise seems like someone/something is trying to break through something	Intensity
Very loud	Intensity
Loud noises	Intensity
New loud sound	Intensity
Loud background noise	Intensity
Similar sound being slowed down and overlapped to create more of a rhythm	Rhythm
Alien background sound	Timbre
Chanting begins	Timbre
New instrument, getting louder	Timbre
Sounds like a spaceship approaching	Timbre
Grunting	Timbre
We encounter some machine	Timbre

Enjoying a similar sound to the 'TARDIS' in 'Doctor Who'	Timbre
Sounds like squirrel and monks praying in background	Timbre
Birds being eaten by big monsters	Timbre
Sounds like a UFO is landing in a jungle	Timbre
Chanting sound begins	Timbre
Bird and wind sound	Timbre
More bird sounds added	Timbre
A new part of the machine is encountered, something involving flow of water	Timbre
A yell from a women	Timbre
Another humanoid sound	Timbre
New voices	Timbre
A new voice was introduced after a distinct introduction to the base sounds	Timbre
Sounds like people talking	Timbre
Noises in background	Timbre
Talking begins	Timbre
People talking in background	Timbre
Sounds like unrecognizable speaking	Timbre
More noise like background speaking people	Timbre
Ladies are mumbling something	Timbre
Noisier, more monsters approaching	Timbre
New sound added	Timbre
Seals?	Timbre
Background noise change, louder	Timbre
The mumbling is getting deeper and dragged out	Timbre
Newspaper blowing	Timbre
Crunching	Timbre
We move on from the machine, walking sounds	Timbre
The 'TARDIS' is gone and has made a shift to a more natural sound	Timbre
Breaths	Timbre
Twigs snapping begins and the rest fades out	Timbre
Bang, buzzing	Timbre
Thud and silence	Timbre
Someone trying to kill a bee	Timbre
Bang, bee	Timbre
Bang	Timbre
Bang	Timbre
Sounds like a bee and something fell	Timbre
A new noise	Timbre
Door shut and mosquito starts buzzing	Timbre
All other sounds have stopped. Can only hear just a fly buzzing	Timbre
There was a noise like a fallen book and the base sounds stopped	Timbre
New sound	Timbre
A book slapped shut and a mosquito got in	Timbre
Fly buzzing	Timbre
Previous sounds gone	Timbre
All sounds stopped and a lonely buzzing sound begins	Timbre
All sound stopped like a door closing, now just feel a bussing noise	Timbre
Buzzing	Timbre
Someone's trying to kill the fly	Timbre
We encounter another machine ramping up, once it gathers steam it seems like some form of artillery	Timbre

New sound introduced	Timbre
Trying to kill the fly	Timbre
Thumping sound	Timbre
Banging noise changes	Timbre
Repetitive banging	Timbre
Door sound has changed and now sounds like a nail gun, mosquito still there	Timbre
The thudding has changed from a book to something falling down a set of stairs	Timbre
Sound is being repeated more often	Timbre
Yet again repetitive banging after fly buzzing sound	Timbre
Frequent thumping sound	Timbre
Banging noise sounds deeper	Timbre
Different type of sound introduced	Timbre
Like a heaving door trying to be broken down	Timbre
Something sliding down strings	Timbre
Banging deeper, man yelling	Timbre
A growl from a human	Timbre
More dramatic sounds	Timbre
Dogs start barking	Timbre
Gun, dog	Timbre
Barking	Timbre
New sounds added	Timbre
Dog barking the mood and tone has changed	Timbre
Dog in background	Timbre
It becomes apparent that the artillery machine is causing explosions in the distance	Timbre
Deep background noise	Timbre
Gun shot	Timbre
Banging/explosions start	Timbre
Echo	Timbre
Someone opening a door to let the bee out	Timbre
New voices	Timbre
Goes from sounding like a war zone to sounding like a wolf howling	Timbre
Dog growling	Timbre
The new clunking sounds stopped with a definitive howl-like sound	Timbre
Previous sounds gone with door opening	Timbre
Animal howling in background	Timbre
Reloading, and time to hear animal calls, possibly those at the receiving end of the machine	Timbre
Many animals howling	Timbre
Change of noise	Timbre
New sound of motor	Timbre
Motorbike	Timbre
Dog sound but different	Timbre
Intro of other sounds has made it feel more organic again	Timbre
Sound of the bike finally getting out and leaving	Timbre
Motorbike	Timbre
Driving away in anger	Timbre
Different motor bike like noise	Timbre
Bird sound in background	Timbre
More background noise	Timbre
Owl hoots	Timbre

Dog barking	Timbre
Goes from forest sounds back to warzone	Timbre
The machine continues	Timbre
Return of explosions	Timbre
Back to dropping sounds	Timbre
Thudding started again	Timbre
More banging	Timbre
The door is trying to be unlocked	Timbre
No more natural noise, I just feel sound that is more man-made	Timbre
Fly is back	Timbre
Buzzing	Timbre
End of explosions	Timbre
The buzzing is back	Timbre
Bug sound prominent	Timbre
Mosquito by itself	Timbre
The clunking stopped with only the buzzing sound remaining	Timbre
Seems like the door is unlocked and the fly entered another room	Timbre
Dead bee	Timbre
Oomph!	Other
(No comment)	Other
Emotional change	Other
Merging	Other
(No comment)	Other
(No comment)	Other
Someone hit something	Other
(No comment)	Other
(No comment)	Other
Change of mood	Other
(No comment)	Other
Joke is still alive	Other
Anger, frustrated	Other
(No comment)	Other
Falling	Other
(No comment)	Other
Fighting	Other
(No comment)	Other
War	Other
A door to the great outdoors was opened	Other
(No comment)	Other
(No comment)	Other

Table D: Qualitative descriptions and designated categories of perceived phrase responses to Eno (1992) 'Francisco'

Qualitative Description Explaining Response	Category
One tone to louder	Intensity
Getting louder	Intensity
The chimes faded for a while then returned	Intensity
The instrument in the background is getting louder	Intensity

The background noise is more louder	Intensity
Louder	Intensity
Music got louder	Intensity
Loud to soft and more background noise	Intensity
The chiming has made the beat go higher changing the rhythm	Rhythm
One of the instruments is speeding up	Rhythm
Like a heart beat or a flutter	Timbre
Piano/chimes begin	Timbre
Alien sound	Timbre
Higher	Timbre
Chimes	Timbre
Whirring	Timbre
A continuous, fast modulating tone as well as a bell-like tone was introduced on top of the drum-like sounds	Timbre
Ray gun	Timbre
Second chime played, sets up new context layer	Timbre
New instruments	Timbre
More drastic alien sound	Timbre
Music tone increased	Timbre
Chiming, higher pitch	Timbre
Piano/chimes begin again	Timbre
Different chimes	Timbre
Third chime played	Timbre
Piercing sound	Timbre
Introduction of piano music has made a shift	Timbre
Deep base note	Timbre
Another flutter	Timbre
Piano/chimes	Timbre
A new instrument	Timbre
New sound introduced	Timbre
Chime-like tones were introduced	Timbre
New sound	Timbre
Sounds like a hang up dial on the phone	Timbre
Higher pitch	Timbre
Chimes	Timbre
Higher pitch chimes	Timbre
Like a triangle	Timbre
Another chime/lower chime reply, the initial chime and lower reply are one event	Timbre
Sounds like a new instrument has been added	Timbre
Creepy organ music	Timbre
Xylophone	Timbre
Intro of a sound has made a shift sounds like old church piano	Timbre
Higher tone	Timbre
Mysterious sound occurs	Timbre
More triangle like sounds	Timbre
Additional, higher noise	Timbre
Creepy owl	Timbre
Higher sound	Timbre
Flutter	Timbre
Changed to a ringing sound not like a phone ring	Timbre
Deep creepy sound	Timbre

Deeper noise	Timbre
Lower pitch noises	Timbre
Background sound	Timbre
Background noise	Timbre
Background noise gone	Timbre
Flutter	Timbre
Chimes/piano	Timbre
New melodic phrase	Timbre
Chime-reply event	Timbre
Background music changed	Timbre
Chimes	Timbre
Creepy piano sound from vampire movies in the castles	Timbre
Chiming, higher noise	Timbre
Change in music tone	Timbre
New instrument added	Timbre
Chimes	Timbre
New instruments	Timbre
Xylophones again	Timbre
Organ again	Timbre
Intro of piano music again over the ringing has made a shift	Timbre
Soothing sound begins in mist of piercing sound	Timbre
Deeper	Timbre
Chiming has stopped. Just a peeping sound	Timbre
Chimes/piano	Timbre
New tones	Timbre
The fast modulating sound became consistent and remained as a single note	Timbre
Switch of sounds	Timbre
Another chime-reply	Timbre
Higher noise	Timbre
Different sound	Timbre
Different chimes	Timbre
Alien strobe sounds	Timbre
A different sound started playing sci-fi sounds. A UFO appeared!	Timbre
Alien chimes	Timbre
Deep alien strobe sounds	Timbre
Change of instruments, noise and tone	Timbre
High to low pitching	Timbre
Similar ringing but a sound you would expect to hear during an old 1950's movie with a UFO scene	Timbre
The fast modulating sound returned as a different pitch	Timbre
Change in tones	Timbre
Creepy piano music again	Timbre
Chiming, higher noise	Timbre
Change in tone of music	Timbre
Chimes	Timbre
Chime reply	Timbre
A weird ringing in the background that comes and goes, softer than the more prominent ring	Timbre
Hang-up dial	Timbre
Flutter	Timbre
Background noise gone	Timbre

Reply occurs without chime preceding it	Timbre
Weary music in the background	Timbre
(No comment)	Other
Ears ringing	Other
(No comment)	Other
(No comment)	Other
Spookier	Other
(No comment)	Other
Irritating	Other
Accidental button press	Other
(No comment)	Other

Table E. Qualitative descriptions and designated categories of perceived phrase responses to Xenakis (1955) 'Metastaseis'

Qualitative Description Explaining Response	Category
Build up	Intensity
Sound of something getting closer	Intensity
Suspense feeling similar to a movie soundtrack	Intensity
Getting louder and more clapping sounds	Intensity
Escalating	Intensity
Louder	Intensity
Getting louder	Intensity
Louder	Intensity
Still intense music but is pushing it overboard in terms of much build up there is	Intensity
Sound peters out	Intensity
Loud	Intensity
The sounds all built up until suddenly they stopped	Intensity
Stopped	Intensity
Dramatic stop	Intensity
Slight pause and then intense	Intensity
Music dropped	Intensity
Momentary stop in blaring	Intensity
Slight pause and back into the suspense again. Getting feelings of a horror jump scary movie	Intensity
Gets aggressive	Intensity
Louder including the background noise	Intensity
Music got lots louder and more intense	Intensity
Cools down for a second	Intensity
Loud sound. Dinging sound began	Intensity
Mood change	Intensity
Louder	Intensity
Frightening sound	Intensity
Increase in intensity	Intensity
Getting louder and more background noise	Intensity
Louder and different sounds	Intensity
Now its aggressive again	Intensity
Dramatic	Intensity
Stressful	Intensity

Soft then loud intense music	Intensity
Increase in intensity again	Intensity
It is getting thrilling	Intensity
More intense	Intensity
Slight pause and more suspense music, the trumpets seem to bridge the gap of suspense music	Intensity
Increase in intensity	Intensity
Really loud aggravating sound appears	Intensity
New sound, loud brass	Intensity
More sounds more stressful	Intensity
Loud new sound introduced	Intensity
Louder and a new instrument	Intensity
Quieter	Intensity
Quieter	Intensity
Quieter	Intensity
The excitement has died down	Intensity
It cools down again	Intensity
The sounds built up again then reduced with only the tuba remaining	Intensity
Sound increased in speed and loudness	Rhythm
The music built up in pace and loudness	Rhythm
Pause	Rhythm
Pause	Rhythm
Pause	Rhythm
Slowing finishing	Rhythm
New drums in background	Timbre
Change of instrument	Timbre
Drumming	Timbre
Guitar plucking introduced?	Timbre
Violin	Timbre
Change in tone	Timbre
Violin led events begin	Timbre
Sounds gone then came back	Timbre
Start of something new	Timbre
Sounds suspenseful and dark	Timbre
New sound	Timbre
Pause, triangle began	Timbre
Bing	Timbre
Stopped, then triangle things	Timbre
New instrument	Timbre
Clock chime	Timbre
Sounds gone chimes	Timbre
A chime	Timbre
New sounds introduced	Timbre
The sounds stopped again with a conclusive bell sound, ready for the next section	Timbre
Suspense as stopped to allow a tattering sound	Timbre
Creepy noise began again, triangle stops	Timbre
Back to evil sound or violin and clapping	Timbre
Return of violins	Timbre
Trumpets join	Timbre
Horn	Timbre
Trumpet only	Timbre

Stops, new instrument	Timbre
Gone	Timbre
Horn	Timbre
Trumpet only	Timbre
Horn or trumpet	Timbre
Circus	Timbre
Trumpets	Timbre
Loud honk	Timbre
Trumpet	Timbre
Higher brass instruments start	Timbre
Trumpets?	Timbre
Horn or alarm - warning sound	Timbre
Horn of a truck	Timbre
Sounds like a truck beep	Timbre
Introduction of horns give a new feeling to this piece of music	Timbre
Toot toot toot toot	Timbre
Repetitive honking	Timbre
The suspense has changed, the trumpets and horns giving the feeling of suspense instead of this drawn-out music	Timbre
Change	Other
Finished	Other
Completely changed mood	Other
Reprieve	Other
Starts again	Other
Mood change	Other

Table F. Qualitative descriptions and designated categories of perceived phrase responses to Beethoven (1804) 'Sonata No. 21 in C major, Op. 53 Waldstein'

Qualitative Description Explaining Response	Category
Louder and faster	Intensity
Faster, aggressive	Intensity
Crescendo	Intensity
Lower notes end event and reset the sequence	Intensity
Softer	Intensity
Calmer	Intensity
Softer music	Intensity
Building	Intensity
Music increased and got louder	Intensity
Louder	Intensity
Louder and faster	Intensity
More intense	Intensity
Speed intensifies then decreases	Intensity
Very loud	Intensity
Crescendo	Intensity
Louder and Fast	Intensity
Low to high	Intensity
Getting softer and quieter	Intensity
Music is a lot softer	Intensity

The sound becomes softer	Intensity
Changed, softer tone	Intensity
Soothing music	Intensity
Louder, faster	Intensity
Getting louder	Intensity
Louder, faster	Intensity
Soothing sound got louder	Intensity
A bit of anger	Intensity
Getting softer	Intensity
Soft to loud	Intensity
Calmer	Intensity
Music sounds got more smooth and relaxed	Intensity
Tempo increased	Rhythm
Increase in tempo and loudness	Rhythm
Pick up of tempo sounds more chaos then rhythm	Rhythm
New pattern emerges, led by the higher notes	Rhythm
Slight pause, speed decreases	Rhythm
Slows down	Rhythm
Separated notes	Rhythm
Slowing down	Rhythm
Beat sounded different	Rhythm
Change of music, slower keys	Rhythm
Slows down for a second	Rhythm
Slow down of the beat creates a more natural feeling	Rhythm
Got slower	Rhythm
Speed increases	Rhythm
Faster paced	Rhythm
Faster again. Moving up the piano	Rhythm
Upbeat with a rhythm, creates a good calm feeling	Rhythm
Faster	Rhythm
Faster keys	Rhythm
Tempo change	Rhythm
Faster again	Rhythm
In beat	Rhythm
Quickening	Rhythm
Faster	Rhythm
Sound becomes faster and louder	Rhythm
Faster and louder	Rhythm
Sound increases	Rhythm
Amount of keys hit decreases, at least that what it sounds like to me	Rhythm
Change in rhythm	Rhythm
Upbeat sound but different feeling than before	Rhythm
Up beat	Rhythm
Slight pause	Rhythm
Slowed down	Rhythm
Change in pace and length of notes	Rhythm
Slower	Rhythm
Slower	Rhythm
Slow tune very vibrant feeling	Rhythm
Change in pattern	Rhythm
High note section starts, rhythm picks up	Rhythm

Quicker melody	Rhythm
Got a lot faster	Rhythm
Faster and higher	Rhythm
Up tempo	Rhythm
Faster and louder	Rhythm
Pattern change	Rhythm
Faster, louder more instruments and noise	Rhythm
Sounds like different beats got introduced	Rhythm
Different rhythm	Rhythm
Increased speed	Rhythm
Faster beat and more exciting	Rhythm
Up beat happy	Rhythm
Very fast	Rhythm
Chaotic sound that includes a rhythm of sorts	Rhythm
Shorter faster melody	Rhythm
Change of verse keys seem faster and lighter	Rhythm
Quicker, scratchy	Rhythm
Getting softer in terms of how many keys the artist is pressing	Rhythm
Pause	Rhythm
Slowed down	Rhythm
Change in pace	Rhythm
Fast but slow	Rhythm
Slowed down	Rhythm
Pause and increase in keys hit	Rhythm
Pause, decrease in speed	Rhythm
Sound increased	Timbre
Higher pitch of the piano is incorporated	Timbre
Harmony	Timbre
Higher	Timbre
The familiar motif that was introduced at the start ends, the other high note section starts again	Timbre
Climbing notes	Timbre
Lower pitch	Timbre
New section, led by the low notes	Timbre
Different sound, lower key	Timbre
Change of music, deeper keys	Timbre
Different melody	Timbre
New sounds	Timbre
Higher key	Timbre
Just the high pitched keys used	Timbre
Different melody	Timbre
Change in pitch	Timbre
From higher scales to lower ones	Timbre
Lower sound prominent	Timbre
So many sounds from the piano create an overly good feeling from a love of classical music	Timbre
Change in the tone of music	Timbre
Different melody	Timbre
Deeper keys progressing into another song	Timbre
New section led by lower notes	Timbre
Higher background noise	Timbre

Low notes drop out, higher notes take over	Timbre
Higher pitch	Timbre
Pitching contour down	Timbre
Higher noise	Timbre
New ticking sound	Timbre
New event of high notes declining scale	Timbre
Different melody	Timbre
New repeated notes	Timbre
Suspense	Other
(No comment)	Other
Dramatic	Other
(No comment)	Other
(No comment)	Other
The previous event has resolved	Other
Mood change	Other
New theme	Other
Different melody	Other
New song	Other
(No comment)	Other
Next section starts after slight pause	Other
Coming back	Other
(No comment)	Other
Mood change	Other
Different melody	Other
(No comment)	Other
(No comment)	Other
(No comment)	Other
New style of music begins	Other
Happy	Other
(No comment)	Other
Sounds like it is ending	Other
Mood change	Other
(No comment)	Other
Leading to new phrase	Other
Different technique	Other
Pleasant	Other
Second phrase	Other
(No comment)	Other
Polite	Other
New phrase	Other
Change in phrase	Other
Drama	Other
(No comment)	Other
(No comment)	Other
Change	Other
(No comment)	Other
Now it sounds like it is ending	Other
(No comment)	Other
(No comment)	Other
The event has resolved	Other
(No comment)	Other

End of a phrase	Other
(No comment)	Other
(No comment)	Other
New phrase	Other
Ending music	Other
(No comment)	Other
Now it sounds like it is ending	Other
Sounding like the end is coming	Other
